# Factors Influencing Properties of Spider Silk Coatings and Their Interactions within a Biological Environment

**DOI:** 10.3390/jfb14080434

**Published:** 2023-08-19

**Authors:** Vanessa T. Trossmann, Sarah Lentz, Thomas Scheibel

**Affiliations:** 1 Chair of Biomaterials, Faculty of Engineering Science, University of Bayreuth, Prof.-Rüdiger-Bormann-Straße 1, 95447 Bayreuth, Germany; vanessa.trossmann@uni-bayreuth.de (V.T.T.); sarah1.lentz@uni-bayreuth.de (S.L.); 2 Bayreuth Center for Colloids and Interfaces (BZKG), University of Bayreuth, Universitätsstraße 30, 95440 Bayreuth, Germany; 3 Bavarian Polymer Institute (BPI), University of Bayreuth, Universitätsstraße 30, 95440 Bayreuth, Germany; 4 Bayreuth Center for Molecular Biosciences (BZMB), University of Bayreuth, Universitätsstraße 30, 95440 Bayreuth, Germany; 5 Bayreuth Materials Center (BayMAT), University of Bayreuth, Universitätsstraße 30, 95440 Bayreuth, Germany; 6 Faculty of Medicine, University of Würzburg, Pleicherwall 2, 97070 Würzburg, Germany

**Keywords:** biomaterials, cell adhesion, film processing, biocompatibility, adjustable coatings, recombinant spider silk proteins, processing parameter

## Abstract

Biomaterials are an indispensable part of biomedical research. However, although many materials display suitable application-specific properties, they provide only poor biocompatibility when implanted into a human/animal body leading to inflammation and rejection reactions. Coatings made of spider silk proteins are promising alternatives for various applications since they are biocompatible, non-toxic and anti-inflammatory. Nevertheless, the biological response toward a spider silk coating cannot be generalized. The properties of spider silk coatings are influenced by many factors, including silk source, solvent, the substrate to be coated, pre- and post-treatments and the processing technique. All these factors consequently affect the biological response of the environment and the putative application of the appropriate silk coating. Here, we summarize recently identified factors to be considered before spider silk processing as well as physicochemical characterization methods. Furthermore, we highlight important results of biological evaluations to emphasize the importance of adjustability and adaption to a specific application. Finally, we provide an experimental matrix of parameters to be considered for a specific application and a guided biological response as exemplarily tested with two different fibroblast cell lines.

## 1. Spider Silk Coatings in Biomaterials Research

**Figure 1:**
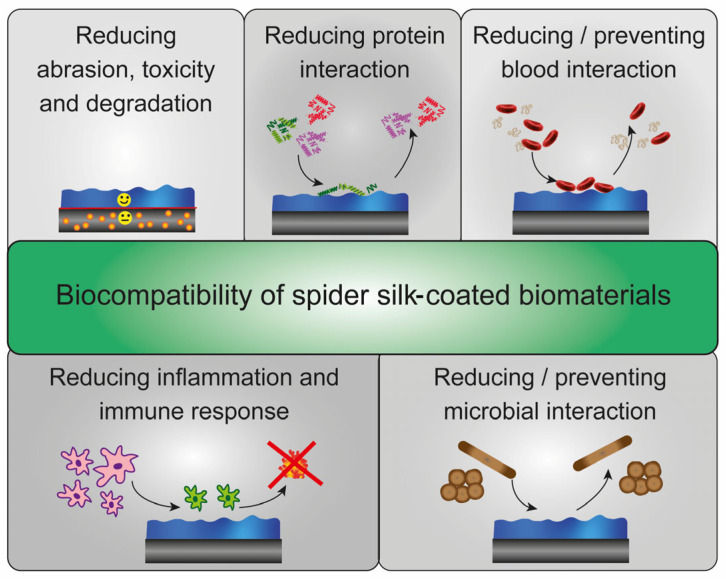
Spider silk coatings enhance the biocompatibility of biomaterials by reducing or preventing the adsorption of proteins, blood coagulation and microbial interaction. Another benefit is the reduction in abrasion, degradation and release of toxic compounds of the underlying material.

Biomaterials are important for human health and quality of life by, e.g., supporting, replacing or restoring injured or destroyed tissues [1,2,3]. Many used materials exhibit excellent application-specific properties but lack biocompatibility and cause immune, inflammatory or allergic responses [1,2,3,4,5]. For instance, polymers’ properties, such as mechanics, biocompatibility or immunogenicity, could change after contact with tissues and body fluids or during degradation and cause unwanted side effects [3,6]. The long-term exposure of metals or metal alloys to multicomponent body fluids containing, for example, enzymes or acids, can also enhance bio-corrosion indicated by the release and dis-solution of metal ions, causing tissue reactions locally or after spreading throughout the body [3,7,8,9,10,11]. Furthermore, metal and polymeric implants often show high bacterial adhesion resulting in many implant-associated infections [12,13,14,15,16]. Otherwise, many metals, ceramics or polymers show limitations concerning important biological and cellular interactions during tissue regeneration and integration [3,17]. Thus, to overcome these issues, multifunctional surface modifications and coatings can be applied to enhance the biocompatibility or bioactivity of such materials [3,5,18,19,20,21,22]. Apart from synthetic coating materials, natural proteins such as silk or the extracellular matrix proteins collagen, laminin or fibronectin show bioactivity and trigger biological responses and cell interactions [23,24,25,26,27,28]. In this context, coatings made of native as well as recombinant spider silk proteins are promising candidates due to their intrinsic properties. They are biocompatible, biodegradable, non-toxic, processable and do not activate immune cells of the inflammatory cascade [26,29,30,31] (Figure 1).

Spider silk coatings can act as a protective, anti-adhesive layer and minimize some adverse effects of commercially available biomaterials [26,29,32,33]. It has been shown that spider silk films demonstrate good in vivo biocompatibility [29,34,35,36]. For instance, spider silk coatings on silicone implants act as a bioshield against the immune system and foreign body responses [29]. Consequently, the coating lowers the risk of fibrous capsule formation and implant rejection reactions [29]. Furthermore, the adsorption of serum proteins, as well as activation of blood coagulation and fibrin formation, are significantly reduced on some spider silk coatings dependent on the spider silk protein used [37,38]. Thus, biomaterial coatings made of spider silk proteins represent a promising method to increase the hemocompatibility of a biomaterial [32,37,38,39,40,41]. Interestingly, several spider silk coatings further show microbe repellence [33,40,41,42,43] or could be modified to combat microbes [36,39,40,41,44,45,46].

**Figure 2:**
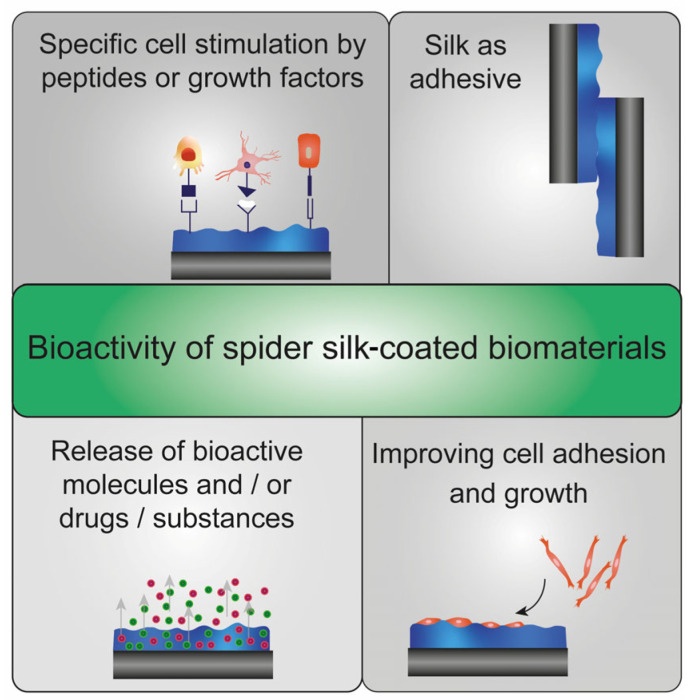
Spider silk coatings enhance the bioactivity of biomaterials by improving cell attachment and growth, explicitly stimulating cells (e.g., through incorporated peptides, growth factors, etc.) or releasing incorporated biologically active substances or drugs. Furthermore, spider silk coatings could be used as an adhesive.

On the other hand, spider silk coatings can be used to increase the bioactivity of biomaterials (Figure 2). Although many native spider silk proteins do not contain cell adhesive protein sequences, they are highly cytocompatible. The introduction of recombinant production strategies allowed application-specific modification of spider silk proteins, such as genetic fusions of cell adhesive peptides, including RGD, KGD or IKVAV, to enable interactions with cell surface receptors (e.g., integrins) [31,42,45,46,47,48,49,50,51,52,53,54,55,56,57]. Such an approach allows the processing of coatings for guided cell adhesion and growth [47,57]. Recently, it could be shown that cell-selective peptides fused with recombinant spider silk proteins led to cell-type specific adhesion on films made thereof [47].

A biomaterials surface coating using spider silk also allows for integrating and releasing biologically active substances and drugs. On the one hand, the bioactive molecules could be chemically or genetically fused to the spider silk protein or added to the casting solvent to be physically incorporated in the resulting film [42,58,59,60]. On the other hand, spider silk films could be processed first and subsequently functionalized [61,62,63]. Recently, recombinant spider silk films were modified with a redox- or a pH-sensitive linker for coupling biologically active anti-cancer drugs [63]. In addition, spider silk materials could be used as adhesive [33]. Lewis and colleagues developed water-based, recombinant spider silk protein coatings, which could also be used as a biological adhesive. They successfully glued materials such as glass, wood, and silicone materials [32,64].

Spider silk coatings allow versatile surface modifications of biomaterials and could be application-specifically adapted. This article highlights important factors that influence the biocompatibility and the bioactivity of recombinant spider silk coatings to achieve surface properties tailored for desired biomedical applications. It is summarized which spider silk proteins, materials, processing techniques and parameters as well as additional factors influence the properties of the resulting spider silk coatings. Apart from an overview of processing parameters, a toolbox of important characterization methods is provided. Furthermore, biological effects on such coatings, including microbial interaction, serum protein adsorption, blood compatibility, immune responses, biomineralization and cell interactions are discussed in detail. Importantly, new data will be presented within a case study, which will clarify how the choice of a spider silk protein, the casting solvent, and the post-treatment method influence the interaction with cells.

## 2. Silk Proteins and Processing Techniques to Gain Spider Silk Coatings

**Figure 3:**
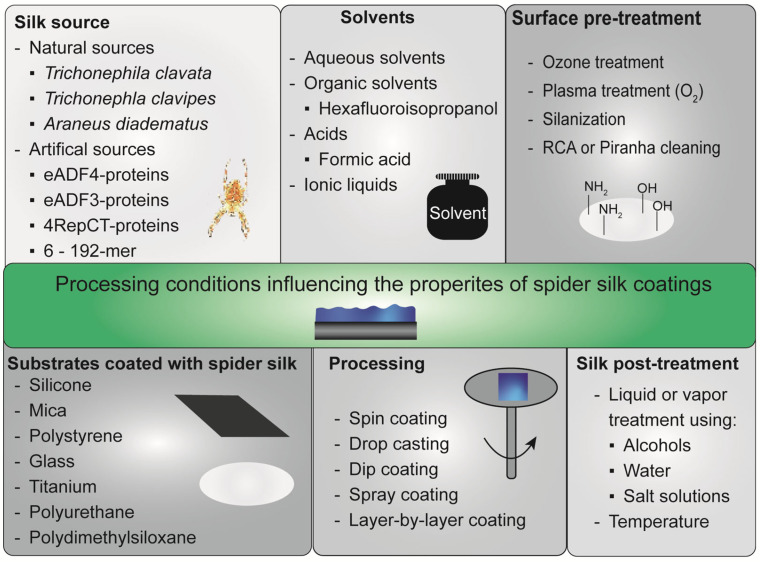
There are several factors influencing spider silk film processing and the resulting film properties. The silk source, used solvents, potential surface pre-treatments, the substrate to be coated, the processing technique and post-treatment methods influence both the processing and the properties of the resulting coating. Abbreviations: RCA (Radio Cooperation of America): cleaning method using hydrogen peroxide, ammonium hydroxide and water (1:1:5).

As mentioned above, spider silk coatings can enhance the biocompatibility and bioactivity of biomaterials. However, this can only be achieved if a homogeneous coating or film is stably deposited on the substrate of interest [53,57,65]. Generally, the diversity of protein sequences as well as processing parameters influences physicochemical film properties and biological interactions [37,66,67,68]. For instance, surrounding water and, thus, relative humidity has a high impact on spider silk film properties [69,70]. Thus, there are several other factors that influence film formation and are relevant to gain a stable coating (Figure 3) [26,71], which will be described in the following paragraphs.

### 2.1. Spider Silk Source

There are two types of spider silk proteins: natural and recombinant ones. Natural spider silk proteins can be received by harvesting spider silk webs and egg sacs or milking spider silk from the appropriate spiders [72,73,74,75,76,77,78,79,80,81,82,83,84,85,86,87,88,89,90,91]. In general, collecting spider silk webs/egg sacs has several disadvantages. For instance, they may contain several non-silk impurities, such as prey leftovers, eggs, pollen or dust, which are also collected. Thus, time-consuming and complex purification processes could be necessary to receive pure spider silk proteins [82,83,91,92,93].

Furthermore, since the spider web comprises different silk types, which are responsible for different features of the web [91,94,95,96,97,98], collected spider web samples could be mixtures of different silk types. Therefore, milking silk fibers directly from the spinning duct of a spider is much better suited to achieve individual spider silks [76,77,78,79,80,81,87,88,89,90,99]. Additionally, extracting the silk type of interest directly from the appropriate silk gland could be an alternative to obtaining spider silk proteins. Although the spider must be sacrificed in this case, the spider silk proteins of interest are received at high purities after gland preparation and extraction [100,101,102,103,104]. However, independent of the collection technique, one major disadvantage of using natural spider silks is that most spiders cannot be farmed on a large scale since they are cannibalistic and territorial. Therefore, the methods for obtaining natural spider silk proteins result in low quantities [91,92,93,105].

In contrast, recombinant protein production techniques allow for overcoming some of these disadvantages. The underlying spider silk genes can be expressed upon genetic engineering in different host organisms, such as bacteria, yeast, insect or mammalian cells, as well as transgenic plants (e.g., tobacco) or animals (e.g., goats) [30,31,91,92,93,105,106,107,108,109,110,111,112,113,114,115,116,117]. Large-scale production of recombinant spider silk is conducted using bacteria (AMSilk, Spiber Inc., Spiber Technologies AB), yeast (Bolt Threads) as well as mammalian cells or transgenic animals (Nexia Biotechnologies) [117]. Prokaryotic bacterial expression systems are highly preferred for recombinant spider silk production since they grow fast, show high productivity and yields, good scale-up options and low production costs [91,93,106,117]. The issues with low expression levels accompanying the highly repetitive sequence of spider silk proteins could be solved by using metabolically engineered *Escherichia coli* and adopted fermentation strategies, but the lack of post-translational modifications remains [91,93,106,108,117,118,119]. However, natural spider silk proteins show post-translational modifications, which might be important for the protein properties, structure, interaction and function [91,106]. For instance, proteomic analysis studies of different spider silk proteins from *Trichonephila clavipes* (*T. clavipes*) identified important post-translational modifications, such as hydroxylation, phosphorylation and methylation, which may be important for the mechano-elastic properties of spider silk [120,121,122]. Some yeast expression systems (e.g., *Pichia pastoris* or *Saccharomyces cerevisiae*) combine fast growth, easy gene expression, protein secretion and distinct post-translational modifications [117,123,124,125], but the post-translational modifications differ from those of higher eukaryotic cells [126,127]. Thus, the host system should be selected according to the needed requirements and envisaged applications, since all expression systems show advantages and disadvantages [91,93,106,108,126].

*T. clavipes* and its spidroins often serve as the natural blueprint for recombinant spider silk production [98,128]. Thus, many other research groups developed recombinant spider silk proteins based on protein sequences of *T. clavipes* over time [30,32,35,36,39,48,52,118,119,129,130,131,132,133,134,135,136,137,138,139,140,141,142,143,144,145,146,147,148,149,150,151]. For instance, Kaplan and colleagues have developed spider silk proteins differing in their MW using one (1mer), six (6mer), 15 (15mer) and up to 192 repeats (fusion of two 96mers) of a consensus sequence, which also contain functional motifs, including peptides for cell interaction, biomineralization or combat microbes [35,36,39,48,49,52,118,119,129,130,131,132,133,134,135,136,137,138,139,152,153,154,155,156,157,158,159]. Interestingly, a fusion protein containing spider silk and mussel foot protein domains has been engineered to enable underwater adhesion [150].

In contrast, we have developed recombinant spider silk proteins based on natural dragline silk proteins of the European garden spider, the *Araneus diadematus* fibroins 3 and 4 (ADF3 and ADF4) [47]. After identifying the amino acid sequence of the natural repetitive core domains, recombinant proteins were developed by multimerizing designed sequence modules to obtain the recombinant spider silk proteins eADF4(C16) (negatively charged) and eADF3(AQ)_12_ (uncharged) [37,43]. Importantly, cloning strategies easily enable modifications; for instance, changing the charge or the molecular weight (MW) of the proteins. Thus, the positively charged eADF4(κ16) [47,57] and the uncharged eADF4(Ω16) [43,47,57], as well as the higher MW variants, such as eADF3(AQ)_24_ [37] or eADF4(C32) [43], have been generated. Furthermore, specific peptide tags could be fused to the recombinant spider silk proteins to enhance cell adhesion (e.g., RGD, KGD, IKVAV) [47,57], biomineralization [55] or to allow site-specific functionalization via engineering cysteine residues [61,63]. In addition, Linder and colleagues used eADF3, eADF4 as well as a part of the native ADF3 sequence as the repetitive block of their recombinant spider silk proteins, which are flanked by different terminal domains [70,160]. Examples of such globular, terminal domains are cellulose-binding modules (CBM), peptide-interacting domains (SPY_C), a highly soluble gamma-crystalline D domain (Crys) and the fibronectin III domain (FN) [70,160,161]. In this context, the terminal domains have application-specific functionalities: thermally stable CBM should enhance cellulose binding [162], FN should increase cell adhesion and tissue regeneration [163] and the SPY_C (SpyCatcher) domains should form an amide bond with an engineered peptide counterpart (SpyTag) enabling adhesion [164,165].

Johansson and colleagues have engineered the recombinant spider silk protein 4RepCT based on one silk protein of *Euprosthenops australis*. 4RepCT contains four repetitions (4Rep) of modules showing poly-alanine- and glycine-rich blocks and a Carboxy-terminal (CT) dimerization domain [166,167,168,169,170,171]. They have also designed modified spider silk proteins carrying cell binding peptides (e.g., RGD, IKVAV and YIGSR) [50,51,172,173,174], antimicrobial peptides (e.g., Lac-peptide (Lactoferricin B) from lactoferrin [175,176] and Mag-peptide from Magainin I [177,178]) [42,45], selective ligand-interaction peptides (e.g., IgG and albumin) [60,179] or entire proteins (e.g., bFGF (basic fibroblast growth factor) to create an artificial extracellular matrix) [45,58,180]. Furthermore, spider silk variants for site-specific conjugation of biofilm- or peptidoglycan-degrading enzymes [46] or bioactive substances (e.g., antibiotics) [181] have been developed. Recently, shorter variants made of two Rep-modules have been developed carrying, for instance, an antimicrobial peptide [182] or both terminal domains [183,184,185,186]. In natural spider silk proteins, the repetitive core domains are also flanked by non-repetitive, globular C- and N-terminal domains, which are essential regulators for storage and fiber assembly [187,188,189,190,191,192,193]. A table with information on recombinant spider silk proteins that are important for this review can be found in the Supporting Information (Table S1).

### 2.2. Spider Silk Purification

Recombinant spider silk proteins are either produced soluble in the cytoplasm, which also allows secretion into the culture media, or aggregated in inclusion bodies, for instance, if bacteria or yeasts are used as an expression host, making a variety of purification methods necessary [124,194]. If proteins are soluble, chromatographical techniques, including ion exchange [107,124,195,196] or affinity chromatography using metal ions (e.g., Ni^2+^, Co^2+^, Zn^2+^, Cu^2+^) [40,48,52,60,70,132,139,146,147,152,160,161,167,197,198] can be used after cell/host disruption. Thermal [43,47,57,107,143,160,161,196,199,200,201] or acidic [194,196,197,199] extraction methods are also used since soluble spider silk proteins are mostly intrinsically unfolded and unstructured. Therefore, they do not unfold upon thermal or acidic treatments, whereas proteins from the host denature aggregate and precipitate [107,194,197,199]. Interestingly, identical proteins could differ in their properties (e.g., secondary structure and zeta potential) after purification depending on the purification strategy (thermal or acidic) used [194]. Furthermore, the properties of assembled spider silk morphologies (e.g., particles, films) could be influenced by the solvent, which is used during purification or processing [194,202]. For instance, hexafluoroisopropanol (HFIP) mainly induces α-helices and random coils, whereas formic acid lead to β-sheets [202].

For purifying soluble recombinant eADF variants (e.g., eADF3 and eADF4) produced in *Escherichia coli*, the bacteria are lysed using a high-pressure homogenization or an ultrasound treatment to disrupt the bacterial cell wall [47,57,70,160]. The soluble proteins are purified using a heat step, ammonium sulfate precipitation, washing steps (e.g., optional dialysis) and freeze-drying [47,57]. Similar purification protocols have been evolved for recombinant spider silk proteins inspired by *T. clavipes* and produced using *Escherichia coli* [107]. However, soluble recombinant rMaSp1 and rMaSp2 spider silk proteins (inspired by *T. clavipes*) produced in the milk of transgenic goats were purified using tangential flow filtration, precipitation, washing and freeze-drying [32,64,140,141,203]. The synthetic chimeric proteins FlYS, FlYS_3_ and FlYS_4_ inspired by *T. clavipes* flagelliform and dragline silk were produced in *Escherichia coli* and purified using ammonium sulfate precipitation, isopropanol, separation, filtration, washing and freeze-drying [32]. Apart from lyophilization, the pure spider silk solution could be frozen in liquid nitrogen and stored at −80 °C [70,160,161]. Upon inclusion body purification, aggregated proteins are often solved in highly concentrated denaturants such as urea and guanidium hydrochloride (GdmHCl) [91,159,204,205,206,207]. For instance, recombinant spider silk proteins, produced in inclusion bodies in yeast, were extracted using 10% lithium chloride solution and 90% formic acid [113,124]. However, since harsh denaturing agents might lead to poor recovery, refolding problems or loss of protein bioactivity, the strategies have to be adopted [91,204,205,206,207].

Hedhammar et al. developed a washing procedure based on Ca^2+^ and EDTA before cell lysis to remove lipopolysaccharides from *Escherichia coli,* decreasing the pyrogenicity of recombinant spider silk proteins [208]. In addition, a triple autoclaving method has been developed to remove temperature-stable endotoxins without changing the mechanical properties of processed materials. In comparison, using dry heat led to dehydration and inferior mechanical properties [203].

### 2.3. Solubilization of Spider Silk Proteins

Depending on the silk source and production process, spider silk proteins are either already solubilized [50,58,70,102,180] or assembled in a solid form (e.g., lyophilized powder, fibers or particles) [43,47,57]. Thus, lyophilized as well as assembled proteins have to be solubilized to generate protein solutions for subsequent processing [26]. If the lyophilized spider silk protein is water soluble, the easiest way to obtain aqueous protein solutions is by dissolving the protein directly in water or aqueous buffers or solutions [32,35,36,37,39,49,52,129,139,209]. For instance, recombinant eADF3(AQ) variants are highly hydrophilic and could be solubilized in various aqueous solutions, including water, buffers or potassium chloride solutions [37]. Recombinant spider silk proteins inspired by *T. clavipes* (rMaSp1, rMaSp2, FLYS variants) were mixed with water, sonicated and heated (microwave) until at least 120 °C was reached [32,203].

If the protein is not water-soluble, it can be dissolved in denaturing agents akin to the procedure of inclusion body solubilization to unfold the proteins, followed by dialysis against aqueous buffer systems to remove the denaturant and obtain aqueous protein solutions [43,59,65,151,210]. Denaturing agents could be highly concentrated chaotropic salt solutions containing lithium bromide (LiBr), lithium thiocyanate (LiSCN), guanidinium thiocyanate (GdmSCN) or GdmHCl [26,120,151,211]. Additionally, highly concentrated urea could act as a denaturant [26,151]. For the subsequent dialysis, water [151] or aqueous buffer systems, including tris(hydroxymethyl)aminomethane (TRIS) [37,63,210], (4-(2-hydroxyethyl)-1-piperazineethane-sulfonic acid (HEPES) [63], ammonium bicarbonate (AmBic) [65,69,212], or potassium chloride (KCl) [37] and potassium phosphate (K-Pi) [213] could be used. Moreover, ionic liquids, such as 1-butyl-3-methylimidazolium chloride (BMIM Cl), 1-ethyl-3-methylimidazolium chloride (EMIM Cl) or acetate (EMIM acetate), and 1-butyl-2,3-dimethyl imidazolium chloride (DMBIM Cl), are possible solvents [26,78,211]. Other solubilization routes are based on fluorinated organic solvents, such as HFIP [47,48,55,69,136,140,152,157,195,213], or (diluted) acids like formic acid [26,34,37,53,57,69,159] or acetic acid [140].

### 2.4. Protein Properties for Processing

Protein properties and their behavior at processing-specific conditions influence their processability and should be evaluated beforehand. In this context, the isoelectric point as well as the pH- and temperature-sensitivity of the soluble protein are important parameters for the protein’s stability in solution [37,43,47]. For successful processing, the spider silk protein should stay in solution at the needed conditions (e.g., solvent, temperature, pH). In this context, the recombinant spider silk protein eADF4(C16) (pI = 3.5) [37,47] represents a highly stable protein since it could be dialyzed in several buffer systems and at different temperatures. In contrast, the uncharged eADF4(Ω16) variant (pI = 7.7) [37,47], differing only in one amino acid in the repetitive module, has to be dialyzed at 4 °C to prevent premature protein aggregation [37,43,47]. Interestingly, uncharged eADF3(AQ) variants (pI = 7.8) are highly water soluble and stable. [37] In some cases, an increase in buffer concentration [63] or changing the ion concentration (e.g., NaCl) stabilizes proteins in solution [158,214]. For instance, Tris buffer is known to interact with glycine-rich peptides and the protein backbone and, thus, stabilizes proteins in solution. This effect is even strengthened with increasing buffer concentrations [215]. Interestingly, the addition of specific salts (e.g., sodium acetate) to the protein solution could influence the hydrophobicity of the resulting films [70]. Small changes, such as exchanges of charged amino acids, led to significant protein property changes, including pH sensitiveness, different solubility or drug interaction [30]. Furthermore, the exposition of charged amino acid residues or functional groups in a film is important for its physicochemical properties and biological effects [66].

### 2.5. Surfaces to Be Coated and Necessary Pre-Treatments

In general, several materials could be coated with spider silk proteins, including polymers, such as polystyrene (PS) [35,36,37,46,47,49,55,61,69,139,141,157,213], silicone [29,32,40,65,136,216,217], polytetrafluoroethylene (PTFE, Teflon) [56,59,65,69,159,218,219], polydimethylsiloxane (PDMS) [43,52,129,140,141,152,154,203,220] and polyurethane (PU) [32,65], metals, including titanium [32] and stainless steel [32,76], composites, such as poly(ethylene terephthalate)/indium tin oxide (PET/ITO) [221], glass [34,37,53,57,69,70], quartz [70,218,222], silica [32,38,137] or mica [37,209]. Interestingly, spider silk coatings could also be applied to biological materials, such as silk fibroin scaffolds [42,45,54], commercial silk sutures (e.g., Perma-Hand^®^) [39] or wood [64], as long as these substrates withstand the casting solvent and coating procedure without degradation. Furthermore, surface-structured substrates showing contours, internal structures or even a 3D topography could be coated with spider silk [32,43,45,54,217].

It is recommended to thoroughly clean or wash the surfaces to be coated beforehand. Simple washing off of hydrophilic and hydrophobic contaminations is achieved by using water and/or (diluted) alcohols, such as ethanol or isopropanol (e.g., 70% or 100% *v*/*v*), and/or organic solvents (e.g., acetone or toluene) [39,53,56,57,222]. RCA cleaning (Radio Corporation of America) is another surface cleaning method that simultaneously represents a pre-treatment with hydroxide ions. It is based on incubation in a mixture of hydrogen peroxide, ammonium hydroxide and water (1:1:5) above 70 °C [37,222]. Hydrochloric acid could be used instead of ammonia [222]. The surface could be used or modified after washing (water) and drying (N_2_). For instance, RCA cleaning could be applied to silica wafers [37] or quartz slides [222]. Applying ozone to the surfaces to increase hydrophilicity is another easy and fast surface cleaning method, representing a pre-treatment [37]. This method could be applied to silica wafers and polystyrene surfaces (e.g., Petri dishes or cell culture plates) [37]. An easy and fast surface pre-treatment is the activation using plasma (e.g., oxygen) to enhance the hydrophilicity and the wetting behavior of a surface [37,40]. However, plasma deposition could also be used to apply a hydrophobic layer on substrates to decrease surface wetting [217]. Plasma treatments could be used for many substrates; for instance, polystyrene or silicone [37,40,217]. Generally, no pre-treatment is necessary for spider silk in highly volatile solvents, such as HFIP, since film formation is quick and complete due to fast solvent evaporation [47,55,69].

Since glass shows a negative surface charge, a coating is only stable in the case of positively or uncharged spider silk variants solved in formic acid and water (5:1) and used due to sufficient interactions between protein and glass [57]. Negatively charged silk forms homogenous films on glass, but they delaminate after contact with liquids (e.g., cell culture media) due to the weak surface adhesion resulting from electrostatic repulsion between silk and glass [53,57]. To address this, silanization using (3-aminopropyl) triethoxysilane (APTES) has to be conducted as surface pre-treatment. Therefore, the washed glass slides are incubated in APTES/ethanol (1:250) at RT for five hours, washed with ethanol and incubated at 70–100 °C to ensure surface modification [53,57]. However, silanization could also be conducted using dichlorodimethylsilane and a desiccator under a nitrogen atmosphere [222].

### 2.6. Coating Techniques

Pouring and casting spider silk solutions are standard techniques for processing films and coatings on many substrates. After solvent evaporation, spider silk films or coatings could be obtained, depending on the used solvent [26,64,70,71,160,220,223]. Reviewed solvents are pure water [32,36] or aqueous buffers [32,59,64,65,69,210,220] and salt solutions [70], acids such as formic acid (sometimes also mixed with water) [37,53,57], or organic solvents, including HFIP [43,47,55,61,69,136,137,152,154,157,216]. Interestingly, the initial formulation and composition of the casting solution could be crucial for the resulting film [32,70]. Since biological responses are mainly driven by interactions with the material’s surface, which are influenced by different properties, such as charge, hydrophobicity, wettability and topography [224,225], homogeneous film/coating formation is essential for comparison and biological evaluation. Due to the simplicity, drop casting [32,37,57,70,136] and pouring [64,140,220] appropriate amounts of spider silk solutions on substrates are frequently used film processing techniques. There, the spider silk solutions could be applied directly to the substrate, and subsequently, the films are formed after solvent evaporation. However, an even distribution of the protein solution must be ensured to guarantee a homogeneous coating. A recent study showed that controlled humidity during solvent evaporation and drying significantly impacts the hydrophobicity and wettability of the resulting film/coating [70]. Interestingly, the application of a structured PDMS stamp during film formation increased the hydrophobicity of the silk film and transferred its surface structures onto the film surface altering the surface wettability and topography [226]. Another film processing method is spin coating. The substrates are fixed on a rotating plate via vacuum pressure, whereas protein solutions are cast on top. Thus, different rotation speeds distribute the applied spider silk solutions consistently with high reproducibility over the substrate surface resulting in thin and homogeneous films [37,216,218,221]. Dip coating can also be used for film formation. The substrates are immersed in the spider silk solution to evenly wet the surface to ensure a homogeneous film after drying and solvent evaporation [32,39,45,53,65,76]. Applying multiple layers leads to thicker and smoother coatings [32]. In one study, Xu et al. used freshly extracted spider silk from major ampullate glands of *T. clavipes* and *Latrodectus hesperus* and prepared films by flattening the silk dope or HFIP solutions on a glass substrate followed by air-drying [102]. Spray coating represents another film-casting method [32]. A gas (e.g., air pressure, N_2_) is applied to a protein solution to generate small droplets sprayed on the substrate of interest. Over time, the droplets on the surface fuse with each other and the solvent evaporates. The even distribution of the droplets is crucial for homogenous film formation [32]. The coating thickness can be adopted by the number of spraying and drying cycles [32]. Interestingly, uniform coatings showing reduced beading during drying could be achieved by applying spray coating before dip coating [32]. Although aerosolized coating techniques are fast, easy and efficient, aqueous coating strategies allow the incorporation of additives and bioactive substances [32].

In general, the film/coating thickness can also be adopted by the protein concentration [32,42,46]. Commonly used concentrations range between 0.01 and 10% (*w*/*v*) [32,47,49,52,59,64,136,139,140,157] but also 15% (*w*/*v*) are reported [195]. In combination with the number of coating cycles or coating formation time, coating thicknesses from the low nanometer range up to several micrometers and even 100 µm can be achieved [32,42,46,59,62,65,219]. Moreover, layer-by-layer coatings can combine different coating techniques and solutions to implement different functions and properties [32,59,65]. Additionally, a washer could be mounted on a glass slide to increase film thickness [102].

### 2.7. Post-Treatment Methods

The sequence of most spider silk proteins contains glycine- and proline-rich amino acid motifs yielding random coil or α-helical secondary structure, as well as poly-alanine stretches, which can form hydrophobic bonds and β-sheet-rich crystallites [227,228]. After film casting, the secondary structure contents of the obtained films differ due to the different casting solvents and methods. Thus, a post-treatment of silk coatings should be conducted to increase the stability against solubilization and degradation [61,65,69,213]. The underlying mechanism is restructuring the spider silk proteins to increase the β-sheet-content of the films [61,139,202,209]. One standard method is using primary alcohols, such as methanol, ethanol and isopropanol [61,69,140,202,210] added either as a pure or diluted alcohol solution [35,36,37,39,49,78,136,137,139,141,154,157,209] to the silk coatings or to a desiccator and connected to a vacuum line to generate a saturated alcohol atmosphere to post-treat spider silk films [47,55,66,152,159]. Highly concentrated potassium and phosphate solutions, (e.g., 1 M potassium phosphate [71,210,213], 1 M potassium chloride [103]) could also be used, even as spray [210]. Furthermore, water steam and high pressure have been used for post-treatment [26,71,76,210]. Such autoclave treatments could also be used to sterilize the films [210] and remove residual solvents [76]. Another easy and gentle post-treatment method is exposing the films to high relative humidity (water vapor treatment) [52,66,154,210] optionally with increasing temperature (water vapor annealing) [66,154]. Importantly, post-treatment using methanol solution seemed to be fast but harsh compared to water-based systems, since crystallinity was increased but functionality (e.g., biomineralization) was inhibited [55,66,154].

Spider silk films cast from HFIP-protein solutions exhibited mainly α-helical or random coil secondary structures due to the fast evaporation of HFIP [71,76,229]. Thus, post-treatment using phosphate ions, primary alcohols or autoclaving is necessary to increase the β-sheet content and to enhance the coating stability [71,76,152,213,229]. Usually, silk coatings out of aqueous solutions show high random coil and α-helical structures and need post-treatment inducing β-sheets [71,139]. However, coatings out of aqueous eADF4(C16) solutions showed high β-sheet content without additional post-treatment even when plasticizers (e.g., glycerol, 2-pyrrolidone) were added [59]. One assumption is that the slow evaporation of the solvent led to controlled self-assembly of the spider silk proteins into β-sheet-rich nano-fibrillar structures [59]. Since spider silk films cast from formic acid already showed a high β-sheet content and water stability, a post-treatment is not absolutely necessary [53,57,69]. Interestingly, post-treatment methods could also alter the wettability and hydrophobicity of the resulting spider silk films [70,152].

## 3. Surface Characterization Methods for Spider Silk Coatings

**Figure 4:**
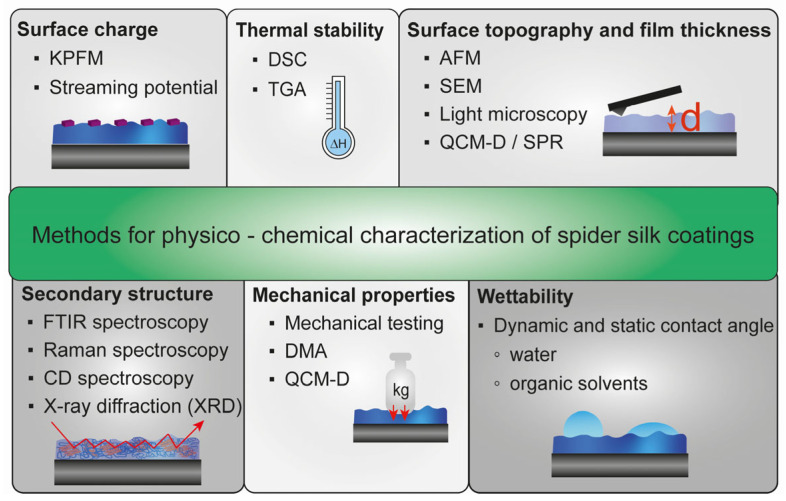
There are several physicochemical characterization methods for analyzing mechanical properties, thermal stability, film thickness, secondary structure, surface charge and the hydrophilicity/wettability of spider silk coatings. Abbreviations: KPFM: Kelvin Probe force microscopy; DSC: differential scanning calorimetry; TGA: thermogravimetric analysis; AFM: atomic force microscopy; SEM: scanning electron microscopy; QCM-D: quartz crystal microbalance with dissipation monitoring; SPR: surface plasmon resonance; FTIR: Fourier-transform infrared spectroscopy; CD: circular dichroism; DMA: dynamic mechanical analysis.

Since a successful spider silk coating is influenced by different factors, coatings must be verified using different physicochemical characterization techniques (Figure 4).

### 3.1. Surface Topography and Thickness of Spider Silk Films

In general, X-ray photoelectron spectroscopy (XPS) and Raman spectroscopy could be used to check that the coating was successful due to the determination of the chemical composition of the material surface [40]. The surface roughness and topography could be analyzed using atomic force microscopy (AFM) [37,46,57,65,69,70,78,129,209,217,222,226] or scanning electron microscopy (SEM) [39,43,69,70,129,131,160,210,217,226,230]. For instance, AFM and SEM revealed that coatings made of 4Rep-CT spider silk proteins comprised self-assembled nanofibrils [42,46,56]. The internal spider silk film structure comprising nanosized filaments could be visualized by SEM of the breaking edges [70]. Välisalmi et al. showed that the relative humidity during film preparation strongly influences the topography and roughness [70]. Interestingly, for different 15mer silk films, a vapor post-treatment and film annealing reduced the surface roughness [152]. Furthermore, eADF4(C16) films cast from an aqueous buffer were rougher than HFIP or formic acid ones [69]. AFM and SEM were also useful tools to visualize and analyze different topographical surface features including grooves, squares, circles or stars on eADF4(C16) films [226].

AFM in the tapping mode [218] and SEM or Field Emission SEM (FE-SEM) [32,40,140,151,231] could be used to evaluate the thickness of spider silk coatings on a biomaterial’s surface. High-precision digital calipers [62] or digimatic micrometers [218] could be used for thicker films. Nilebäck et al. used acoustic QCM-D and optical SPR real-time monitoring to record spider silk coating formation from an aqueous spider silk solution on a gold sensor surface. Even after initial protein adsorption, the coating thickness increased due to continuous protein adsorption and protein-protein interactions and remained stable after washing [42].

### 3.2. Mechanical Properties of Spider Silk Films

Thin spider silk coatings are usually bonded with the underlying material and could be bent without cracks or deformation due to their flexibility [29,32,65]. Usually, thin spider silk coatings do not influence the mechanical properties of the bulk underlying material; for instance, silk sutures [39]. But the mechanical properties of silk films are influenced by the film thickness [59,210]. However, the mechanical properties of spider silk films are significantly lower compared to that of fibers as shown for materials made of natural *Trichonephila clavata* silk [76]. There are several methods to determine the mechanical properties of spider silk films; for instance, using a rheometer [40,77]. One study developed an in vitro flow system on a rheometer to simulate the in vivo conditions of a catheter and showed that a spider silk coating on silicone catheters is stable under blood flow conditions [40]. Mechanical measurements could also be conducted using a tensile tester equipped with a capacity load cell. Therefore, the films were cut in strips, fixed and exposed to force measurements [59,62,76,131,140,151,159,161,210,218,219]. For instance, dry eADF4(C16) films cast from aqueous solutions exhibited impressive elastic moduli (5500 MPa) and tensile strength (81 MPa) but only low elongation (1.8%) [59]. Additionally, eADF4(C16) films cast from HFIP on Teflon and post-treated with methanol, showed slightly different mechanical properties (elastic modulus (3300 MPa), tensile strength (52 MPa), elongation (1.8%)) [62]. Generally, post-treatment increased β-sheet content and consequently the elastic moduli and strength of the films while decreasing the elasticity [71]. Adding plasticizers, such as glycerol or 2-pyrrolidone, increased elongation but led to a loss of stiffness [59,71,210]. Compared to Nylon 66, poly(l-lactic acid) (PLA) and polyethylene films, spider silk films were stiffer but also more brittle [59]. Incorporating other polymers (e.g., polycaprolactone (PCL) or thermoplastic polyurethane (TPU)) decreased Young’s Modulus and tensile strength of eADF4(C16) significantly but increased the elongation at break [62]. The mechanical properties could also be determined using force deformation measurements and bulging [231]. Free-standing, wet FN-4RepCT membranes showed a strain of 223%, a stress of 4.7 MPa and a toughness of 5.2 MPa [231]. Dynamic mechanical analysis (DMA) could also be performed [69,141]. The mechanical properties of eADF4(C16) films increased after methanol post-treatment independent of the used casting method due to the increase in β-sheet content [69]. Compared to free-standing and hydrated MaSp2 films, MaSp1 ones (both *T. clavipes*) showed higher mechanical properties due to the different sequences [141]. Interestingly, the mechanical properties of films made of autoclaved, endotoxin-reduced spider silk films were comparable to those made of non-treated spider silk proteins [203]. Furthermore, spider silk films could also be stretched inside a methanol [219] or isopropanol:water bath (80:20) to increase mechanical properties [140,203]. Using the dissipation to frequency ratio of the quartz crystal microbalance with dissipation (QCM-D) monitoring data allowed the evaluation of the viscoelastic properties of spider silk coatings. The initial layer of 4Rep-CT variants is rigid, but the continuously assembling silk layers displayed more viscous properties [42]. AFM (contact mode) was conducted to measure force curves to evaluate the viscoelastic properties of film surfaces [151,209]. For instance, 6mer spider silk films showed a lower Young’s modulus than 6mer-BSP films functionalized with a peptide from bone sialoprotein (BSP) [209]. Interestingly, it was shown recently that blending spider silk proteins with natural or synthetic polymers could increase the mechanical properties of the resulting materials [232]. In this context, composite films made of a recombinant ADF3 variant and poly-alanine exhibited higher tensile strength and toughness than silk films [219].

### 3.3. Thermal Stability of Spider Silk Films

There are several experimental techniques for analyzing the thermal properties of silk proteins [233]. One method to determine the thermal properties and stability of spider silk coatings is differential scanning calorimetry (DSC). There, films are loaded in metal pans (e.g., aluminum or gold), heated up or cooled down to an appropriate temperature in a nitrogen atmosphere, whereas thermograms are recorded [59,78,131,202,210]. For instance, non-post-treated eADF4(C16) coatings cast from aqueous solutions displayed a typical bimodal melting curve (thermal decomposition). The glass transition temperature (T_g_) was determined around 214 °C, whereas the endothermal peak was around 336 °C indicating high thermal stability of spider silk coatings, which was also preserved when plasticizers (e.g., glycerol, 2-pyrrolidone) were added [59]. However, it is assumed that the film-casting method influences the structural film matrix composition leading to different thermal properties [210].

Thermogravimetric analysis (TGA) using a thermobalance can also be used to determine thermal stability [62,78,131,202,218]. There, samples are filled in aluminum oxide pans or ceramic crucibles and heated up (25 °C to 800 °C) in a nitrogen atmosphere to record thermograms [62,131,202]. Non-post-treated recombinant eADF4(C16) films cast from HFIP on Teflon exhibited a faster decomposition than methanol post-treated films [62]. The thermal stability of spider silk films (e.g., made from eADF4 variants) is an important prerequisite for biomedical applications since some sterilization methods (e.g., steam sterilization) require high temperatures [59].

### 3.4. Secondary Structure of Spider Silk Films

There are several experimental methods for characterizing the secondary structure of silk proteins [233]. Fourier-transform infrared (FTIR) spectroscopy allows for determining the secondary structure content of spider silk coatings by analyzing the amide I protein band [218,229,231]. Therefore, measurements are conducted using an FTIR spectrometer or a Hyperion microscope with an attenuated total reflectance (ATR) module/objective [35,40,49,52,57,59,76,129,139,159,209,210] or a Germanium crystal [53,69,219]. In addition, grazing angle attenuated total reflection (GATR) FTIR spectroscopy could be performed with a mercury cadmium telluride (MCT) detector [37,218]. Two-dimensional infrared correlation spectroscopy is also an option to investigate conformational changes [103]. Interestingly, spider silk proteins differing in their amino acid composition could differ in their secondary structure contents [37,71,229]. Furthermore, the initial secondary structure of silk films is highly dependent on the casting solvent used [69]. When comparing water vapor annealing with liquid methanol post-treatment, methanol induces higher β-sheet contents, whereas more random coils remain after water vapor annealing. However, silk protein-dependent differences were visible [154]. Interestingly, structural properties differ significantly on hydrophilic and hydrophobic substrates independent of the used solvent. On hydrophilic substrates, β-sheets are exposed to the surface (hydrophobic), whereas on hydrophobic substrates the helical and random coil conformation is surface-exposed (hydrophilic) [69].

X-ray diffraction (XRD) also enables characterizing the secondary structure content of silk films [62,131,140]. Non-post-treated eADF4(C16) films showed X-ray diffraction peaks at around 14° and 19°, indicating an α-helical structure, whereas methanol post-treated films had peaks at 17°, 20°, 24°, and 32° verifying β-sheet content [62]. The crystallinity resulting from β-sheets could also be determined using wide-angle X-ray scattering (WAXS) [76] or diffraction (WAXD) [219]. Autoclaving *Trichonephila clavata* films cast from HFIP led to plastification by water and increased the crystallinity from 6.9% to 14.1% [76]. Raman spectroscopy is another method to determine the secondary structure of spider silk films [40,102,140,195]. Raman spectra displayed that non-post-treated films made from natural *T. clavipes* dopes showed β-sheets, whereas *L. hesperus* films mainly consisted of random coils and α-helices [102]. Furthermore, the secondary structure of silk coatings and films could be determined using circular dichroism spectroscopy [70,218,222]. Recombinant Crys-ADF3-Crys films exhibited β-sheet content independent of the relative humidity during film preparation (RH: 35% and 80%) [70].

### 3.5. Wettability of Spider Silk Films

The measurement of static contact angles, mainly water contact angles, characterizes the wettability and hydrophobicity of the surface of a material [32,40,52,56,62,65,69,70,152,218]. Apart from the commonly used water, dimethylformamide [52,152], ethylene glycol [52,152] and milk [231] were used to analyze the wettability and surface energy of spider silk films. In addition to static measurements, dynamic contact angles could be determined using a continuous water flow at an optical tensiometer [70].

Several investigations showed that spider silk coatings increase the wettability and hydrophilicity of commonly used biomaterial surfaces, such as glass, polystyrene, catheter materials (silicone, PU, PTFE), steel or titanium [32,37,40,47,56,57,69]. Thus, most of the studies mentioned above categorize spider silk film surfaces as hydrophilic. However, spider silk coatings could increase the hydrophobicity of mica [37] or glass [69,70] surfaces, which could be influenced by the solution conditions (e.g., presence of salts) and the surrounding humidity (RH) during film formation [70]. In this context, the WCA increased from 20° (up to 45% RH) to around 120° (above 65% RH), which could be decreased to 87° using a methanol post-treatment [70].

Water contact angle measurements could also evaluate spider silk surface modifications. For instance, the hydrophobicity of eADF4(C16) films was increased, if a (structured) PDMS stamp was applied during film formation (above 80°) [226]. A C-terminal modification with the silica-binding peptide R5 significantly affected the contact angle of 15mer-films after post-treatment (without R5: 116° vs. with R5: 75°) [152]. Furthermore, the hydrophobicity of eADF4(C16) films (52°) increased upon hydrazine modification (61°) and subsequent para-dimethylaminobenzaldehyde (DMAB) (67°) coupling. However, the comparability of surface wettability of spider silk coatings is limited by the fact that, depending on the study, contact angles have been determined at different time points; for instance, directly after contact [63], after three [62], ten [47,57] or over 90 s [70].

### 3.6. Surface and Zeta Potential of Spider Silk Films

There are several methods to determine the surface or zeta potentials of spider silk coatings. For instance, Kelvin probe force microscopy (KPFM) has been conducted to determine the surface potential of a spider silk coating [37]. The streaming or zeta potential of the coatings could be measured using an electrokinetic measuring device [37,59]. Electrokinetic measurements revealed variations for coatings made of eADF4(C16) depending on the processing conditions. Although eADF4(C16) films (formic acid, silica wafer, O_2_-pre-treatment, MeOH vapor post-treatment) showed an isoelectric point (IEP) of 3.9 similar to that of the soluble protein [37], Agostini et al. determined an IEP of 5 for eADF4(C16) coatings (Tris buffer, Teflon, no post-treatment) [59]. Furthermore, we analyzed the streaming (zeta) potential of differently charged eADF spider silk coatings (formic acid, silica wafer, O_2_-pre-treatment, MeOH vapor post-treatment), showing that the streaming potential of uncharged and positively charged coatings could differ from the IEP of soluble proteins and the theoretical IEP due to the different hydrophobicity as well as the influence of charged amino acid residues, especially in case of mainly uncharged proteins [37].

## 4. Biological Effects on Spider Silk Coatings

**Figure 5:**
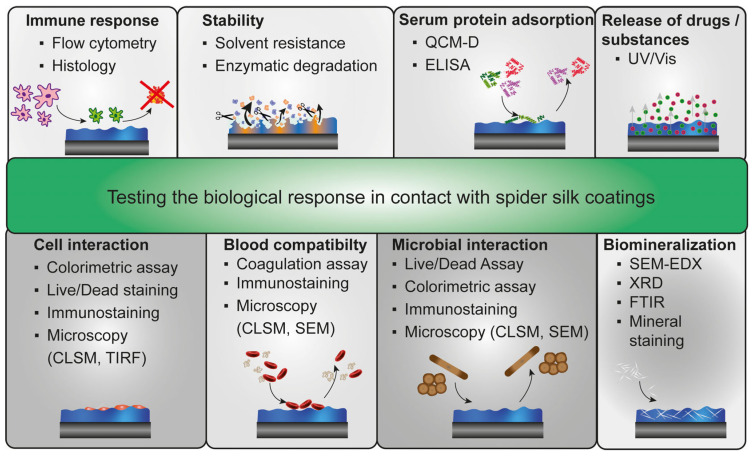
There are several characterization methods for analyzing biological responses (e.g., immune response, coating stability, serum protein adsorption, blood compatibility, microbial or cell interaction, biomineralization and release of substances) in contact with spider silk coatings. Abbreviations: ELISA: enzyme-linked immunosorbent assay; CLSM: confocal laser scanning microscopy; TIRF: total internal reflection fluorescence microscopy; EDX: energy dispersive X-ray spectroscopy; XRD: X-ray diffraction.

Spider silk coatings can be used for many applications, including tissue engineering and biomedical applications, making the requirements and the biological evaluation versatile (Figure 5). Coatings made of recombinant spider silk proteins represent an easy and chemical-free functionalization strategy to adjust biomaterial and implant surfaces [37,42,47,50,57]. For instance, 4RepCT coatings could be applied to common implant materials, such as hydroxyapatite and gold surfaces, or polystyrene surfaces, a commonly used material in cell culture to bridge differences in mechanical and chemical properties of implant materials and surrounding tissue [42].

### 4.1. Immune Response against Spider Silk Coatings

Most (recombinant) spider silk proteins are non-inflammatory and non-toxic. In contrast, medical-grade silicone implants usually show to some extent, increased immune responses, inflammation, fibrillar capsule formation, and finally, rejection [29]. A thin recombinant eADF4(C16) spider silk coating significantly reduced these foreign body responses and ensured implant incorporation in rats. Therefore, eADF4(C16) was dialyzed against Tris/HCl buffer. Ethanol-washed silicone surfaces were dip-coated with spider silk solution and post-treated using potassium phosphate (1 M). In vitro studies revealed that monocytes could proliferate on eADF4(C16) coatings but did not differentiate into CD68-positive macrophages, which is important for immune responses. Furthermore, coated silicone implants were tolerated for up to 12 weeks in rats with reduced fibrous capsule formation, inflammation and immune response. Thus, these spider silk coatings significantly increase the biocompatibility of silicone implants [29].

Subcutaneous implantation of films made of 6mer spider silk [35,36] and 6mer functionalized with antimicrobial hepcidin [36] and a peptide from bone sialoprotein (BSP) [35] in mice revealed good biocompatibility of silk coatings in vivo. Although flow cytometry of isolated and stained cells from the implant site displayed the presence of different immune cells, histological analyses indicated a low and mild inflammatory reaction to implanted spider silk films without fibrous capsule formation [35,36]. Spider silk membranes made of pNSR16 and pNSR32 showed moderate inflammation after implantation, which decreased during wound healing indicating good biocompatibility [34].

### 4.2. Solvent Stability and Enzymatic Degradation of Spider Silk Coatings

The sufficient stability of spider silk coatings in different solvents is essential for biomedical applications dealing with different body fluids. An easy way is incubating the spider silk films in the solvent of interest; for instance, water, PBS, urea (8 M), GdmHCl (6 M) or GdmSCN (6 M), optionally using slight shaking [59,131,210,217,229]. After defined incubation intervals, the protein concentration in the solvent was determined using UV-Vis photo spectroscopy [59,210]. For instance, some protein was released in the first hour for non-post-treated coatings cast from aqueous eADF4(C16) solutions, but the coating remained stable afterward [59]. Another study used eADF3(AQ)_12_, eADF4(C16) and blend films cast out of HFIP and showed that films without post-treatment were soluble in all tested solvents. The post-treatment using methanol led to an increase in stability, especially against water. However, protein-sequence-dependent differences in solubility were detectable [229]. For instance, post-treatment using methanol is also required for 6mer-based spider silk proteins to prevent the films from dissolving in an aqueous environment (e.g., body fluids, media) [35,36,139]. Interestingly, spider silk films prepared at an ambient humidity of 35% were water-stable, whereas the same films prepared at 80% RH partly dissolved after incubation with water droplets [70]. QCM-D measurements also verified that recombinant 4RepCT coatings were stable during washing with different solutions, including PBS, sodium hydroxide (0.1 M and 0.5 M), hydrogen chloride (0.1 M and 0.5 M), ethanol (20% and 70%) and Tris buffer [42].

Dependent on the application, the degradation behavior of spider silk films is crucial. For instance, drug depots should degrade faster than an implant coating [37]. Both the charge as well as the amino acid composition of the protein seemed to influence the degradation of spider silk coatings by protease mix PXIV and collagenase [37]. eADF4-based spider silk protein films showed a relatively slow degradation behavior induced by proteases (e.g., protease mix PXIV from *Streptomyces griseus* or collagenase) [37,65]. Interestingly, positively charged eADF4(κ16) films exhibited a higher degradation rate compared to negatively charged eADF4(C16) and uncharged eADF4(Ω16), with more than 60% of films made of recombinant eADF4 variants remaining after 15 days [37]. Uncharged eADF3(AQ) variants quickly degraded within two days since eADF3(AQ) variants contain more recognition sequences for these enzymes [37]. Thus, eADF3 variants are promising candidates for drug delivery applications, whereas eADF4 variants could be used for stable coatings necessary for biomedical applications, such as in catheters, medical devices or implants [37]. Moreover, the resistance of eADF4(C16) films against trypsin or elastase degradation could be enhanced by adding biopolymers such as PCL or TPU [62]. Additionally, recombinant 6mer spider silk coatings could also be degraded by protease PXIV [39]. Composite films made of a water-dissolved 72mer sMaSp1 spider silk protein and collagen were degraded by collagenase within 24 h [131]. Enzymatic degradability could also be characterized using chymotrypsin [69,151]. Thereby, non-crystalline, amorphous spider silk parts are degraded faster than remaining β-sheets, which could be verified using FTIR spectroscopy and SEM [69].

### 4.3. Serum Protein Adsorption on Spider Silk Surfaces

Serum protein adsorption on biomaterial surfaces is a natural but, at the same time, critical process since the initially formed protein layer guides subsequent immune and inflammatory responses. Coatings made of recombinant eADF4 and eADF3 variants differing in amino acid composition and surface charge were analyzed regarding the interaction and adsorption of essential blood proteins [37,38]. QCM-D analyses revealed that the total protein adsorption of essential blood components, such as immunoglobulin G (IgG), human serum albumin (HSA) and fibrinogen (Fib), was influenced by the amino acid composition and the charge of the spider silk coating. In general, uncharged eADF3 variants showed less protein adsorption than eADF4 variants, even the uncharged eADF4(Ω16) [37]. Hence, increased serum protein adsorption arose from the higher hydrophobicity of eADF4 variants [37], a common fact also seen for fibrinogen and, generally, hydrophobic surfaces [234,235,236]. However, when comparing only eADF4 variants differing in their charge, it could be shown that positively charged eADF4(κ16) surfaces clearly supported serum protein interaction more compared to negatively charged eADF4(C16) and uncharged eADF4(Ω16). Generally, not the amount of adsorbed fibrinogen but the restructuring, conformational changes and accessibility of a cryptic site of fibrinogen are critical for blood coagulation and thrombotic fouling. An enzyme-linked immunosorbent assay (ELISA) revealed that positively charged eADF4(κ16) also induced the restructuring of fibrinogen, whereas other variants showed less impact on conformational changes [37]. Thus, to minimize the adsorption of blood serum proteins and restructuring of fibrinogen, spider silk coatings combining positive surface charge and high hydrophobicity should be avoided [37].

### 4.4. Blood Interaction and Hemocompatibility of Spider Silk Surfaces

Blood coagulation after contact with a biomaterial’s surface is critical for future applications in the body. Coatings made of recombinant eADF4 and eADF3 variants were analyzed regarding blood coagulation. It could be shown that the surface charge but not the amino acid sequence influenced blood coagulation. Positively charged eADF4(κ16) surfaces triggered interaction with blood components, clotting and fibrin network formation [37,38]. In contrast, negatively charged eADF4(C16) and uncharged eADF4(Ω16), as well as uncharged eADF3(AQ)_12_ and eADF4(AQ)_24_ coatings, showed no blood coagulation nor fibrin network formation [37,38]. Different biomaterial substrates, such as steel, titanium and silicone, were coated with a recombinant rMaSp1/rMaSp2 spider silk protein layer. Using spray-coating and drying cycles, aqueous silk solutions, optionally mixed with 1% (*w*/*v*) heparin, were applied and dried. The coatings were incubated with *Caprine* blood, supplemented with 0.3% (*w*/*v*) sodium citrate to prevent blood clotting. Afterwards, 50 mM calcium chloride was added to track blood clotting. After washing and drying, thrombotic fouling was analyzed. Although unmodified substrates showed high accumulation of blood components, heparin-functionalized spider silk coatings significantly reduced blood clotting and thrombotic fouling. Interestingly, compared to blank materials, spider silk coatings without heparin already reduced blood interaction and clotting significantly, verifying the inherent ability of these spider silk surfaces to prevent and reduce thrombotic fouling [32]. Another recombinant, heparin-binding MaSp2-spider silk protein S4H4 showed anticoagulant properties and good hemocompatibility in presence of heparin. Although the heparin-binding was evaluated using an affinity dot blot and ELISA, the anticoagulation properties were verified using an activated partial thromboplastin time assay assessing the conversion of prothrombin to thrombin [41]. Interestingly, a S4H4 coating still showed anticoagulant properties after exposure to bacteria [40]. In addition, 6mer- and antimicrobial 6mer-HNP1-coatings displayed reduced hemolytic activity and red blood cell damage compared to reference materials (e.g., silicone, rubber or steel) [39].

### 4.5. Biomineralization of Spider Silk Films

For bone and dental tissue engineering, biomineralization is a prerequisite for success. Recombinant spider silk proteins have been modified with silica-binding peptides (e.g., R5, A1) [66,136,152,154,157], hydroxyapatite binding domains (e.g., VTK-peptide) [52], peptides from bone extracellular matrix proteins (e.g., bone sialoprotein (BSP, sialo) or osteopontin (osteo)) [35,49,55,209], the C-terminal domain of dentin matrix protein 1 (CDMP1) [137] or whole proteins (e.g., silaffin) [66] to enhance mineralization [30,105]. The mineral deposition on these spider silk surfaces could be carried out in simulated body fluid [55,137] and other defined salt solutions [49,52], tetramethoxysilane in phosphate buffer [136], tetraethyl orthosilicate solution [66,152,154] or in cell culture media (e.g., DMEM) containing many different salts at different concentrations [49,55]. Biomineralization could be analyzed via (ATR-)FTIR [49,55,137], X-ray diffraction (XRD) [52,55], contact angle measurements [52,55], SEM connected to an elemental analysis (SEM-EDX) [49,52,55,137,152,154,157], transmission electron microscopy (TEM), Alizarin Red S [52,55,152] or Von Kossa staining [35]. SEM images revealed that non-treated as well as post-treated R5-functionalized 6mer and 15mer, as well as A1-6mer films, exhibited enhanced silicification. At the same time, spider silk variants without peptides contained less or no silica structures on their surfaces [66,136,152,154,157]. Interestingly, water-based 15mer-films significantly enhanced silicification compared to HFIP-based ones. Furthermore, water annealing at RT resulted in high biomineralization compared to methanol treatment or water annealing at higher temperatures [66]. After incubation in SBF, 15mer films functionalized with CDMP1 showed enhanced calcium and hydroxyapatite nanocrystal deposition verified by SEM-EDX, FTIR and TEM [137]. Films made of another fusion protein, 6mer-BSP, induced the nucleation of (tri-)calcium phosphates and hydroxyapatite leading to mineral deposition in vitro [49]. After implanting these films subcutaneously in mice, a Von Kossa staining of histological tissue sections after six weeks revealed calcium deposition and thus, biomineralization in vivo [35]. In addition, VTK-modified 15mer spider silk films increased crystalline calcium phosphate and hydroxyapatite deposition indicated by XRD, SEM-EDX and Alizarin Red S analysis [52]. Neubauer et al. biomineralized coatings from modified eADF4(C16) variants cast from HFIP without any post-treatment in SBF and DMEM. FTIR, XRD and SEM-EDX verified that the formed crystals contain calcium, phosphate, sodium and chloride. Although aqueous conditions led to a partial film solubilization, simultaneously, water induced a slow and gentle post-treatment of spider silk proteins allowing mineral deposition on the film surface. In contrast, post-treatment of these coatings using liquid methanol for one hour dramatically reduced mineral deposition in both fluids indicating that protein restructuring during methanol post-treatment led to reduced accessibility of the fused peptide modification afterward [55]. Reduced biomineralization (silicification) was also shown for different 6mer films [154]. Interestingly, defined biomineralization came along with enhanced cell adhesion and proliferation on peptide-modified variants [49,52,55].

### 4.6. Cell Interaction with Spider Silk Surfaces

Most natural spider silk proteins, and thus, their recombinant counterparts, do not contain cell-interaction sites in their primary amino acid sequence. Therefore, they do not support cell adhesion and growth. For instance, murine fibroblasts showed round morphology (SEM) and no growth (DNA amount) on films made of natural *Trichonephila clavata* dragline silk [76]. Furthermore, coatings made of recombinant eADF4(C16) and eADF4(Ω16) variants do sparsely support cell interaction [47]. Many different cell types, including fibroblasts [37,47,65], cardiomyocytes [47,53,57], neuronal cells [37,47,65], myoblasts [47,65], keratinocytes [47,65] and cancer cells (e.g., HeLa) [47] could not interact. In contrast, positively charged eADF4(κ16) spider silk films supported interactions with cells, such as cardiomyocytes [47,53,57], fibroblasts, myoblasts and neuronal cells [47,65].

However, it could be shown that cell interaction with spider silk materials could be influenced by many factors, including the post-treatment, the substrate surface pre-treatment and the processing technique. Fibroblasts adhered to eADF4(C16) cast from HFIP on Teflon and post-treated using methanol (liquid), although morphological differences were visible. Compared to the treated cell culture plate, 72% of the Balb cells were attached but exhibited a round cell shape after six hours of incubation [62]. We analyzed 18 different spider silk films partially functionalized with cell-binding peptides regarding primary cell adhesion of eleven different cell types. All films were processed from HFIP on non-treated polystyrene surfaces and post-treated using ethanol vapor overnight to ensure comparability. It could be shown that eADF4(C16) and eADF4(Ω16), as well as eADF4(C16)-RGE and eADF4(C16)-GFPGER provide non-cell adhesive spider silk surfaces. In contrast, positively charged eADF4(κ16) surfaces supported cell interactions without stimulating peptide and only by charge interactions. Thus, specific interactions could be better evaluated on negatively charged eADF4(C16)- and uncharged eADF4(Ω16)-based variants [47]. In one study, the polystyrene plates were pre-treated using ozone to ensure a homogenous spider silk coating after formic acid evaporation and post-treated using 75% (*v*/*v*) ethanol. Primary cell attachment was enhanced on positively charged eADF4(κ16), but long-time incubation showed that cells could not stay or proliferate [37]. In contrast, cardiomyocytes could attach to eADF4(κ16) surfaces cast from formic acid on negatively charged glass slides and exhibited synchronous beating over long-time incubation [53,57].

Non-post-treated eADF4(C16) coatings carrying peptides from bone proteins were analyzed regarding biomineralization and MC3T3 mouse pre-osteoblast interaction using a cell titer blue assay and fluorescence staining of cell compartments. Adhesion and proliferation were increased on as-cast and pre-mineralized films as well as on a linear gradient film (made of eADF4(C16) and eADF4(C16)-Osteo), where more cells attached with increasing content of the mineralization variant eADF4(C16)-Osteo after 11 days [55]. Additionally, osteoinductive VTK- and R5-modified 15mer as well as 6mer-BSP spider silk films supported the adhesion, proliferation and osteogenic differentiation of human mesenchymal stem cells with and without pre-mineralization [49,52,152,157]. Furthermore, osteogenesis after eight weeks was verified using Alizarin Red S staining, detecting deposited calcium and enhanced bone-sialoprotein formation indicated by immunostaining [52]. Furthermore, 6mer and 15mer variants functionalized with antimicrobial peptides allowed interaction and proliferation of mammalian cells including fibroblasts and osteosarcoma cells [39,129]. RGD-modified 15mer films promoted the adhesion and proliferation of human mesenchymal stem cells [48]. The 6mer-films functionalized with fibronectin type II enhanced adhesion of skin fibroblasts if blended with a silk-elastin-like protein [159]. Another recombinant analog of *T. clavipes* supported the growth of fibroblasts over four days [196].

Moreover, a fusion protein consisting of spider silk and elastin enhanced the adhesion and growth of human chondrocytes [111]. Interestingly, recombinant MaSp1 films (*T. clavipes*) exhibited a suitable amino acid composition (GRGGL motif) and substrate stiffness to support neuronal growth, axon extension and network connectivity [141].

Interestingly, recombinant 4RepCT-films promoted mammalian cell interaction, including neural stem cells [168] and primary human dermal fibroblasts [230], even without a biochemical modification. RGD-modified FN-4RepCT-spider silk coating increased cell attachment and subsequent proliferation of keratinocytes [45,56], dermal fibroblasts [45], endothelial [45,174], smooth muscle [174], bone [56] and mesenchymal stem cells [174] up to seven days. Additionally, keratinocytes could attach and grow on both sides of free-standing membranes made of FN-4RepCT, which allowed free diffusion of molecules (e.g., Dextran, BSA and blood plasma proteins) but hindered permeation of gold nano- and polystyrene-microparticles [231]. FN-4RepCT coatings also enhanced bone cell adhesion and proliferation even if linked biofilm- or peptidoglycan-degrading enzymes were present [46]. Moreover, bFGF-4RepCT coatings supported human umbilical vein endothelial cell interaction, which could be increased by blending with FN-4RepCT [45]. Histological stainings and gene expression studies revealed that FN-4RepCT coatings on fibroin scaffolds also promoted wound healing, tissue remodeling, re-epithelialization and vascularization in vivo in rats [54]. Other spider silk membranes made of pNSR16 and pNSR32 also supported wound healing of rats in vivo verified by enhanced synthesis of bFGF and collagen formation [34].

Apart from biochemical interaction ligands, topographical surface features could also influence cellular responses to a surface [237,238,239,240]. A recently published study showed that topographical surface patterns on eADF4(C16) films enabled selective cell adhesion to a usually non-cell-adhesive material. The surface indentations showing different shapes and dimensions, including grooves, circles, squares and stars, served as anchoring points, supported cell alignment and enabled contact guidance [226].

### 4.7. Microbial Interaction with Spider Silk Surfaces

Microbial interaction differs depending on the spider silk used and must be evaluated individually for all spider silks [241]. Although one study showed increased bacterial adhesion and growth in natural spider silk [242], several other studies indicated that selected spider silks could be microbe repellent [243,244,245,246,247,248]. Kumari et al. analyzed recombinant eADF3 and eADF4 variants differing in amino acid composition and charge regarding their microbial interaction compared to *Bombyx mori* (*B. mori*) silk fibroin coatings on PDMS surfaces. A structural model for microbial interaction of spider silk surfaces and repellence was developed based on hydrophobic patches. *B. mori* fibroin coatings contain large hydrophobic patches supporting microbial attachment and biofilm formation. In contrast, coatings made of uncharged eADF3(AQ)_12_ and negatively charged eADF4(C16) and eADF4(C32)-NR4 showed homogeneously distributed, small hydrophobic patches, which are too small for microbial interaction and, thus, showing microbial repellence. However, uncharged eADF4(Ω16) coatings, differing only in one amino acid in the repetitive module compared to eADF4(C16), could not wholly inhibit microbial interaction since the hydrophobic patches are densely packed and not separated by charge repulsion as in the case of eADF4(C16) [43].

However, recombinant spider silk proteins could be modified with specific, cationic, antimicrobial peptides to actively combat microbes [36,39,40,41,44,45,46,129,139]. The 4RepCT and 4RepCT-Mag (Mag-peptide from Magainin I [177,178]) coatings on polystyrene disks were incubated in *Staphylococcus aureus* bacterial culture for 24 h or 48 h, washed by gently dipping the samples in peptone water and examined by Live/Dead staining. Compared to pure polystyrene, unmodified 4RepCT coatings already showed a decreased number and viability of bacteria, but the presence of an antimicrobial Mag-peptide had a significantly larger effect [42]. Antimicrobial Mag-4RepCT and Lac-4RepCT (Lac-peptide (Lactoferricin B) from lactoferrin [175,176]) coatings reduced biofilm formation and growth of *Pseudomonas aeruginosa* and *Staphylococcus epidermidis* [45]. Additionally, 4RepCT spider silk coatings modified with biofilm- or peptidoglycan-degrading enzymes reduced the adhesion of *Staphylococcus aureus* [46].

Interestingly, a recombinant, heparin-binding MaSp2-spider silk protein S4H4 was designed to act as an antimicrobial coating due to the intrinsic antimicrobial efficacy of the heparin-binding peptide. The antimicrobial properties against *Escherichia coli* [41] and *Staphylococcus aureus* [40] were evaluated using zone of inhibition, crystal violet, bioluminescent ATP or colony assays and displayed reduced bacterial adhesion and growth compared to uncoated controls [40,41].

Recombinant 6mer and 15mer spider silk were genetically modified with the antimicrobial peptides hepcidin, human neutrophil defensin 1 (HNP-1), 2 (HNP-2) and 4 (HNP-4) [36,39,129,139] affecting Gram-negative and Gram-positive bacteria [39,129,139] and fungi [129]. Radial diffusion assays revealed that functionalized 6mer and 15mer proteins functionalized with antimicrobial peptides displayed antimicrobial activity against several microbes including *Escherichia coli*, *Staphylococcus aureus*, *Enterococcus faecalis*, *Pseudomonas aeruginosa*, *Bacillus pumillus* and *Candida albicans*, and no cytotoxic effects against mammalian cells [39,129,139]. Thereby, bioengineered 6mer proteins showed higher antimicrobial activity compared to modified 15mer variants [129]. Blend films made of silk fibroin and 6mer or 6mer-HNP-1 significantly reduced adhesion, viability and biofilm formation of *Staphylococcus aureus* compared to pure silk fibroin films [129]. Especially, 6mer-HNP1-coatings reduced adhesion and biofilm formation of *Escherichia coli* and *Staphylococcus aureus* on commercial silk sutures verified using SEM, live/dead staining and colony-forming assays [39].

In another study, antimicrobial substances, including kanamycin, gentamicin, tetracycline, ampicillin and chloramphenicol, were solved in aqueous recombinant rMaSp1/rMaSp2 solutions (dragline silk proteins from *T. clavipes*) and processed into films on different substrates, such as steel, titanium and silicone, using dip coating. Microbes, including *Staphylococcus aureus*, *Serratia marcescens*, *Escherichia coli*, *Pseudomonas aeruginosa* and *Candida albicans* were incubated with spider silk and antibiotic-coated biomaterials for 24 h. The functional coatings inhibited the growth of these microbes, indicated by inhibition zones without microbe growth around the applied materials on the appropriate culture plates. Importantly, *Pseudomonas aeruginosa* showed intrinsic resistance against some antibiotics except gentamicin [32].

### 4.8. Release of Substances from Spider Silk Films

Spider silk films were remotely loaded after film processing by incubating the assembled film in an appropriate drug solution [62]. For this loading method, spider silk coatings and drugs of interest should exhibit the opposite charge [59]. In one study, recombinant eADF4(C16) films cast out of HFIP on Teflon and post-treated using methanol (liquid) were incubated (15 min) in a saturated PBS-solution containing the polycationic low molecular weight model drugs methyl violet or athacridine lactate [62]. Both model drugs were constantly and slowly released over one month. The release could be moderately accelerated in the presence of elastase and trypsin and significantly decreased by adding biopolymers such as PCL and TPU. However, the maximum drug load decreased with increasing biopolymer content [62].

Herold et al. [63] described another method for subsequent spider silk film modification with drugs for targeted delivery and trigger-controlled release of drugs in cancer therapy and treatment of acute or chronic diseases. There, spider silk films (e.g., eADF4(C16), ntag^Cys^-eADF4(C16), ntag^Cys^-eADF4(κ16)) cast out of HFIP on polystyrene and post-treated using ethanol were chemically functionalized to enable first a covalent coupling of model drugs and afterward a redox- or pH-triggered release of these substances. The triggered release could be achieved by decreasing the pH for coupled para-dimethylaminobenzaldehyde (DMAB) or by enhancing the concentration of reducing agents for bound 5-thio-2-nitrobenzoic (TNB) acid [63]. The redox- and pH-triggered system is designed to release the active ingredients only in the tissue/cells of interest, whereas conventional therapies administer drugs mainly intravenously over the whole body [63,249].

A third alternative is directly loading substances by solving the drug and mixing spider silk and drug solutions before film casting, as mentioned before, for antibiotics-loaded spider silk films. The one-step process as well as the available amount of incorporated substance are benefits of this method [59]. Coatings were processed from aqueous eADF4(C16) solutions, modified with the model substances lysozyme, FITC-dextran and bovine serum albumin (FITC-BSA) or the model drugs tetracaine hydrochloride and paracetamol using drop casting on Teflon. Interestingly, film processing and homogenous distribution were successful for neutrally charged paracetamol or FITC-dextran and negatively charged FITC-BSA, whereas protein aggregation or turbid films (inhomogeneous drug distribution) were obtained for positively charged lysozyme and tetracaine hydrochloride, respectively. Thus, soluble spider silk protein and drug should not display opposite charges for direct loading to minimize electrostatic interactions [59]. Interestingly, the release kinetics of drugs from eADF4(C16) films could be adjusted by adding glycerol, which increased the hydrophilicity of silk coatings, or using layer-by-layer coatings [59]. Water-based eADF4(C16) coatings without using organic solvents or additional post-treatments allow for a high coating stability and incorporation of sensitive therapeutic biologicals [59].

## 5. Influence of Solvent, Silk Protein and Post-Treatment Method on Cell Adhesion on Spider Silk Films

**Figure 6:**
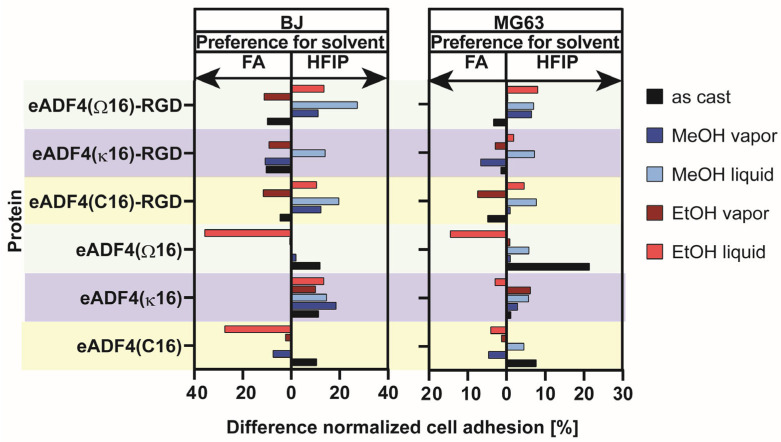
Influence of casting solvent, spider silk variant and post-treatment on cell adhesion. Normalized adhesion [%] of human BJ skin fibroblasts and human MG63 bone fibroblasts on spider silk films cast out of formic acid (FA) or HFIP and post-treated differently after four hours. (*n* = 3) The difference in normalized cell adhesion was calculated by subtracting the cell titer blue values (Figures S1 and S2) of same films (FA and HFIP) from each other. Thus, the size of the bar indicates the relative differences in cell adhesion and shows how the solvent and the post-treatment method could promote cell interaction with an appropriate spider silk variant.

Here, a systematic study with six different eADF4 variants was performed to analyze the influence of the solvent, the protein variant and the post-treatment method (Figure 6 and Figure S1) on cell interaction. The comparability of cell culture studies is essential for several reasons. When comparing cell culture studies, reproducibility must be guaranteed to ensure similar experimental conditions, validity and reliability. Since inter-study comparisons are complex, systematic studies must identify commonalities and differences. Another critical point is the translation to in vivo studies. In vitro cell culture studies are often used as a preliminary step, allowing comparison to extrapolate the findings.

Here, eADF4 proteins were either solved in HFIP or formic acid at similar concentrations and cast on ozone-pre-treated polystyrene cell culture surfaces. Ozone pre-treatment was necessary to ensure homogenous film formation out of formic acid [37]. After evaporation of the solvent, each spider silk film comprised 0.5 mg spider silk protein per one cm^2^ film area, independent of the spider silk variant and solvent used, as described previously [37,47,57]. Further, the influence of different post-treatment methods on film properties and cell interaction was analyzed using ethanol and methanol as solution (liquid) or saturated atmosphere (vapor). Two different human cell lines were chosen to display cell-type-specific differences. The normalized cell adhesion (determined using a cell titer blue assay) is visualized in bar diagrams (Figures S2 and S3). To highlight the best solvent/post-treatment combination for enhanced cell interaction, the cell titer blue assay values for each recombinant spider film cast out of HFIP for human BJ skin and MG63 bone cells were subtracted from those of formic acid. This relative distance is shown as a bar diagram (Figure 6). It indicates the preference of the cells (human BJ skin or MG63 bone cells) to the casting solvent/post-treatment method for each spider silk variant individually. For each spider silk protein and post-treatment method, the bar indicates for which solvent (HFIP or formic acid) a higher number of living cells could be detected after four hours.

For instance, eADF4(κ16) always showed higher cell adhesion when the coating was cast out of HFIP. eAD4(Ω16) coatings cast out of formic acid and post-treated using liquid ethanol exhibited the most significant difference in cell adhesion for both cell types. These results also indicated that the cell adhesion is strongly guided by using a specific combination for film processing and post-treatment method, even if the used spider silk variant usually does not promote cell interaction (e.g., eADF4(C16), eADF4(Ω16)). Interestingly, the variants carrying the integrin binding sequence RGD also showed solvent and post-treatment-dependent preferences in cell interaction. This led to the assumption that the post-treatment has a significant impact on the orientation of the RGD on the film surface and makes the peptide more or less accessible for cells.

## 6. Materials and Methods

### 6.1. Spider Silk Protein Production

The recombinant spider silk proteins are based on the repetitive core domain of one naturally occurring spider dragline silk protein, *Araneus diadematus* fibroin 4 (ADF4), of the European garden spider. Negatively charged eADF4(C16), positively charged eADF4(κ16) and uncharged eADF4(Ω16) were engineered by multimerizing a consensus module 16 times [47,57]. Furthermore, a cell-binding RGD-peptide was C-terminally fused to the repetitive spider silk proteins to enhance cellular interactions [47,57]. eADF4(C16) was purchased from AMSilk GmbH (Munich, Germany), whereas the other variants were produced using fed-batch fermentation and purified as described previously [47,57].

### 6.2. Spider Silk Film Casting

Recombinant spider silk proteins were dissolved in either HFIP or formic acid (VWR, Germany) at a concentration of 10 mg/mL overnight (HFIP) or for one hour (formic acid). Before film casting, 96-well non-treated polystyrene tissue culture plates (Nunc, Thermo Scientific, Bremen, Germany) were pre-treated using UV/ozone (PDS-Pro Series, Novascan, Ames, Boone, IA, USA) for 10 min as described previously [37] to ensure a homogenous film formation after solvent evaporation. Films were produced by drop casting 16 µL of the appropriate silk solutions into the wells to obtain films exhibiting 0.5 mg silk protein per one cm^2^ film area as described previously [37,47,57].

### 6.3. Post-Treatment of Spider Silk Films

Since high proportions of crystalline β-sheets make spider silk films water-stable, an appropriate post-treatment using primary alcohols was conducted to induce the restructuring of the silk proteins [47,61,69]. Here, different post-treatment methods were conducted to analyze their influence on cell interaction. On the one hand, film surfaces were covered with ethanol or methanol solution and incubated overnight at RT to allow complete evaporation of the alcohol solution. On the other hand, films were incubated in ethanol or methanol vapor atmosphere inside a desiccator overnight.

### 6.4. Cell Adhesion on Spider Silk Films

Cell adhesion studies were conducted using human skin BJ fibroblasts (CLR-2522, ATCC, Manassas, VA, USA) and human bone MG-63 osteosarcoma fibroblasts (CRL-1427, ATCC, USA) in a cell culture incubator (HERACell 150i, Thermo Fisher Scientific, Dreieich, Germany) at humidified conditions containing 5% CO_2_ at 37 °C. BJ skin fibroblasts were cultured in Eagle’s minimum essential medium (EMEM, Sigma-Aldrich, Darmstadt, Germany) supplemented with 10% *v/v* fetal calf serum (FCS, BioSell, Feucht, Germany), 1% *v/v* GlutaMax (Gibco, Thermo Fisher Scientific, Dreieich, Germany) and 0.1% *v/v* gentamycin sulfate (Sigma-Aldrich, Darmstadt, Germany). MG-63 bone fibroblasts were cultured in EMEM supplemented with 10% *v/v* FCS, 1% *v/v* GlutaMax, 1% *v/v* non-essential amino acids (Sigma-Aldrich, Darmstadt, Germany) and 0.1% *v/v* gentamycin sulfate. Sub-culturing of both cell lines was conducted using trypsin. An automated cell counter (TC20, Bio-Rad Laboratories, Feldkirchen, Germany) and trypan blue (Sigma-Aldrich, Darmstadt, Germany) was used to determine cell numbers and viability.

Before use in cell culture, non-treated and post-treated spider silk films were sterilized using UV light for 40 min and washed using 1× phosphate-buffered saline (PBS, Sigma-Aldrich, Germany). For analyzing cell adhesion on silk surfaces, 10,000 BJ skin fibroblasts and 15,000 MG-63 bone fibroblasts were seeded in 96-well plates and incubated for four hours. Treated 96-well cell culture plates (Nunclon, Thermo Scientific, Germany) served as controls. Afterward, culture media and non-attached cells were soaked off. Cell adhesion was analyzed using a cell titer blue assay (alamarBlue, Promega, Walldorf, Germany) since cells can metabolize resazurin (blue) to resofurin (pink). Therefore, each 96-well was incubated with 150 µL of 10% *v/v* cell titer blue reagent in appropriate cell culture media for 150 min. Samples without cells served as blank controls to detect the self-degradation of resazurin. Afterward, 100 µL of the supernatant was transferred to a black 96-well plate (Nunc, Thermo Scientific, Dreieich, Germany). The fluorescence of resofurin was measured at a wavelength of 590 nm using an automated plate reader system (Mithras LB940, Berthold Technologies, Bad Wildbad, Germany). The values from the cell titer blue assay were subtracted from each other to obtain a preference for each combination of solvent, cell line and recombinant spider silk variant and visualized as relative differences in a bar chart (Figure 6, Figures S2 and S3).

## 7. Conclusions

**Figure 7:**
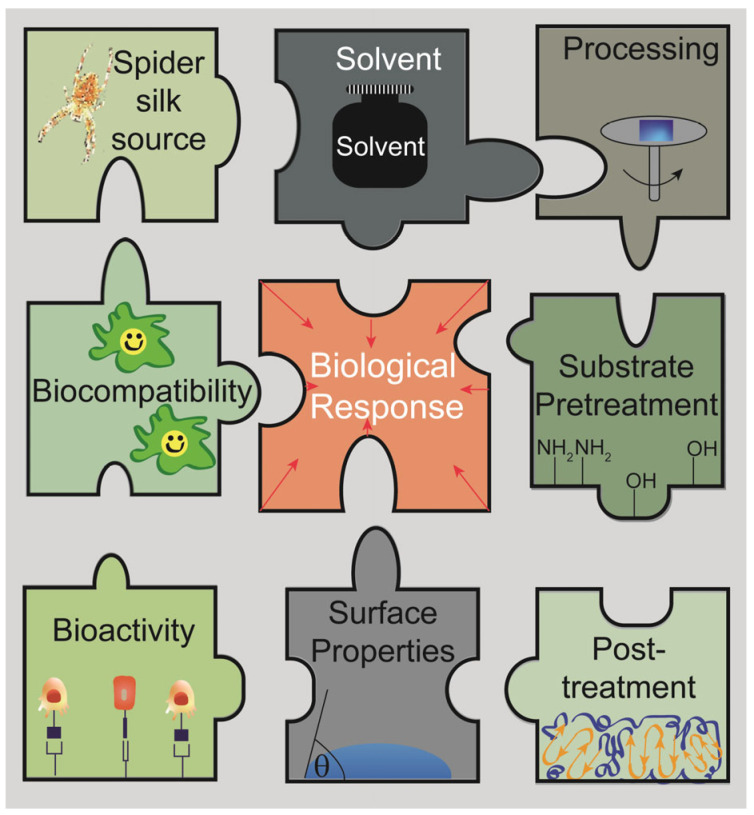
Factors influencing properties of recombinant spider silk coatings and their biological responses. The biological response in contact with a spider silk coating is influenced by various factors, including the silk source, solvent, processing technique, pre- and post-treatments, wettability, bioactivity, and biocompatibility. Importantly, knowing which factors have which effects helps to utilize and design coatings for specific applications.

In conclusion, a direct comparison of studies on spider silk coatings and their interactions within a biological environment is difficult due to the multitude of factors that influence spider silk film/coating properties (Figure 7). However, many studies presented in this review showed that the protein folding, film properties and biological interactions could be adopted by tuning the protein characteristics and controlling the process parameter.

First of all, the chosen silk protein has an impact on the resulting film properties due to the different amino acid compositions, size and polarity of its sequence. In this context, the recombinant production of spider silk proteins is highly advantageous since it allows the generation of adapted spider silk proteins specifically modified for intended applications. Modifications on the genetic level enable adjustments of molecular properties; for instance, changes in molecular weight, charge, or hydrophobicity. In addition, the spider silk processing techniques are responsible for the thickness and structure of the coating. The used materials, including solvents, substrates, and pre-and post-treatments, further alter the film/coating properties due to their chemical properties and composition. One must be aware that substrate pre-treatments and spider silk post-treatments are necessary to ensure a stable film/coating formation. However, at the same time, they influence the chemical interactions and, thus, the arrangement and structure of the proteins of the coating. Thus, the properties of coatings made of the same protein can differ due to the usage of different solvents, substrates, or treatments. Regarding possible medical applications, spider silk coatings have to be evaluated regarding biological responses, such as interactions with cells, microbes, proteins or blood. This can provide insights into biocompatibility, stability, degradation behavior and tissue ingrowth of a coated implant. After identifying valuable and critical spider silk properties and process parameters, these necessary key factors could be combined to generate perfectly suited and adapted spider silk coatings. Taken together, the huge benefits of spider silk coatings, especially those made of recombinant proteins, are their tunability and adaptability regarding a wanted biological response. The properties of the final coating are determined by many different factors, which should be selected and combined to design a surface, providing a wanted biological response.

## Data Availability

The data that support the findings of this study are available from the corresponding author upon reasonable request.

## Supplementary Materials

The following supporting information can be downloaded at: https://www.mdpi.com/article/10.3390/jfb14080434/s1, Table S1: showing the recombinant spider silk proteins, the spider species, the production host, the amino acid sequence of the repetitive module and the associated reference; Figure S1: scheme of the sample matrix; Figure S2: cell adhesion of MG63 on films made of eADF4 variants (a) as cast or post-treated with (b) MeOH vapor, (c) MeOH liquid, (d) EtOH vapor, (e) EtOH liquid; Figure S3: cell adhesion of BJ on films made of eADF4 variants (a) as cast or post-treated with (b) MeOH vapor, (c) MeOH liquid, (d) EtOH vapor, (e) EtOH liquid.
