# Supplementary material for: Factors Influencing Properties of Spider Silk Coatings and Their Interactions within a Biological Environment
Source: J Funct Biomater. 2023 Aug 19;14(8):434. doi: 10.3390/jfb14080434 (PMC10455157; doi:10.3390/jfb14080434)
Supplement: Supplementary file 1 [file jfb-14-00434-s001.zip › jfb-2517964-supplementary.pdf]

Supplementary Materials

Factors Influencing Properties of Spider Silk Coatings and Their Interactions within a Biological Environment

Vanessa T. Trossmann [1], Sarah Lentz [1] and Thomas Scheibel [1,2,3,4,5,6,*]

1 Chair of Biomaterials, Faculty of Engineering Science, University of Bayreuth, Prof.-Rüdiger-Bormann-Straße 1, 95447 Bayreuth, Germany; vanessa.trossmann@uni-bayreuth.de (V.T.T.); sarah1.lentz@uni-bayreuth.de (S.L.)
2 Bayreuth Center for Colloids and Interfaces (BZKG), University of Bayreuth, Universitätsstraße 30, 95440 Bayreuth, Germany
3 Bavarian Polymer Institute (BPI), University of Bayreuth, Universitätsstraße 30, 95440 Bayreuth, Germany
4 Bayreuth Center for molecular Biosciences (BZMB), University of Bayreuth, Universitätsstraße 30, 95440 Bayreuth, Germany
5 Bayreuth Materials Center (BayMAT), University of Bayreuth, Universitätsstraße 30, 95440 Bayreuth, Germany
6 Faculty of Medicine, University of Würzburg, Pleicherwall 2, 97070 Würzburg, Germany
* Correspondence: thomas.scheibel@uni-bayreuth.de

Table S1. Summary of recombinant spider silk proteins, spider species, production host and respective amino acid sequence.

Protein	Spider species	Production host	Amino acid sequence	Reference
eADF4(C16)<br><br>eADF4(C32)	*Araneus diadematus*	*E. coli*	C- module repeated 16 or 32 times<br>GSSAAAAAAAASGPGGYGPENQGPSGPGGYGPGGP<br>+ cell adhesion peptides (e.g. RGD, IKVAV)<br>+ biomineralization peptides<br>+ cystein-containing peptide (e.g. ntagCys)	[1-7]
eADF4(κ16)	*Araneus diadematus*	*E. coli*	κ-module repeated 16 times<br>GSSAAAAAAAASGPGGYGPKNQGPSGPGGYGPGGP<br>+ cell adhesion peptides (e.g. RGD, IKVAV)<br>+ cystein-containing peptide (e.g. ntagCys)	[1,3-6]
eADF4(Ω16)	*Araneus diadematus*	*E. coli*	Ω-module repeated 16 times<br>GSSAAAAAAAASGPGGYGPQNQGPSGPGGYGPGGP<br>+ cell adhesion peptides (e.g. RGD, IKVAV)	[1-4]
eADF3(AQ)$_{12}$<br><br>eADF3(AQ)$_{24}$<br><br>eADF3 with functional domains	*Araneus diadematus*	*E. coli*	A and Q module repeated 12 or 24 times<br>(A) GPYGPGASAAAAAAGGYGPGSGQQ<br>(Q) GPGQQGPGQQGPGQQGPGQQ<br><br>Domains: cellulose-binding domains (CBM), peptide interacting domains (SPY_C), gamma-crystalline D domain (Crys), fibronectin III domain (FN)	[1,2]<br><br>[8-10]

4RepCT	*Euprosthenops australis*	*E. coli*	GGSGNSGIQGQGGYGGLGQGGYGQGAGSSAAAAAAAAAAA<br>AGGQGGQGQGGYGQGSGGSAAAAAAAAAAAAAAAAGRGQG<br>GYGQGSGGNAAAAAAAAAAAAAAAAGQGGQGGYGRQSQGA<br>GSAAAAAAAAAAAAAAAGSGQGGYGGQGQGGYGQSSASASA<br>AASAASTVANSVSRLSSPSAVSRVSSAVSSLVSNGQVNMAALP<br>NIISNISSSVSASAPGASGCEVIVQALLEVITALVQIVSSSSVGYIN<br>PSAVNQITNVVANAMAQVMG (contains C-terminal domain)<br>+ cell adhesion peptides (e.g. RGD, IKVAV)<br>+ antimicrobial peptides (e.g. Lac and Mag)<br>+ specific ligand-interaction peptides (e.g. IgG and albumin)<br>+ entire proteins (e.g. FGF)	[11-36]
2RepCT			GNSGRGQGGYGQGSGGNAAAAAAAAAAAAAAAAGQGGQGG<br>YGRQSQGAGSAAAAAAAAAAAAAAAGSGQGGYGGQGQGGY<br>GQSGNSVTSGGYGYGTSAAAGAGVAAGSYAGAVNRLSSAEAA<br>SRVSSNIAAIASGGASALPSVISNIYSGVVASGVSSNEALIQALLE<br>LLSALVHVLSSASIGNVSSVGVDSTLNVVQDSVGQYVG<br>+ N-terminal domain	[37-41]
rMaSp1	*Trichonephila clavipes*	*Transgenic goats*	QGAGAAAAAAGGAGQGGYGGLGGQGAGQGGYGGLGGQGA<br>GQGAGAAAAAAAAGGAGQGGYGGLGSQGAGRGGQGAGAAA<br>AAAGGAGQGGYGGLGSQGAGRGGLGGQGAGAAAAAAAAGG<br>AGQGGYGGLGNQGAGRGGQGAAAAAAGGAGQGGYGGLGS<br>QGAGRGGLGGQGAGAAAAAAGGAGQGGYGGLGGQGAGQG<br>GYGGLGSQGAGRGGLGGQGAGAAAAAAAAGGAGQGGLGGQ<br>GAGQGAGASAAAAGGAGQGGYGGLGSQGAGRGGEGAGAA<br>AAAAGGAGQGGYGGLGGQGAGQGGYGGLGSQGAGRGGLG<br>GQGAGAAAAGGAGQGGLGGQGAGQGAGAAAAAAAGGAGQ<br>GGYGGLGSQGAGRGGLGGQGAGAVAAAAAGGAGQGGYGG<br>LGSQGAGRGGQGAGAAAAAAAGGAGQRGYGGLGNQGAGRG<br>GLGGQGAGAAAAAAAAGGAGQGGYGGLGNQGAGRGGQGAA<br>AAAGGAGQGGYGGLGSQGAGRGGQGAGAAAAAAAVGAGQE<br>GIRGQGAGQGGYGGLGSQGSGRGGLGGQGAGAAAAAAAGGA<br>GQGGLGGQGAGQGAGAAAAAAAGGVRQGGYGGLGSQGAGR<br>GGQGAGAAAAAAAGGAGQGGYGGLGGQGVGRGGLGGQGAG<br>AAAAGGAGQGGYGGVGSGASAASAAASRLSSPQASSRLSSAVS<br>NLVATGPTNSAALSSTISNVVSQIGASNPGLSGCDVLIQALLEV<br>VSALIQILGSSSIGQVNYGSAGQATQIVGQSVYQALG	[42-48]
rMaSp2			PGGYGPGQQGPGGYGPGQQGPSGPGSAAAAAAAAAAAGPGGY<br>GPGQQGPGGYGPGQQGPGRYGPGQQGPSGPGSAAAAAAAGSG<br>QQGPGGYGPRQQGPGGYGQGQQGPSGPGSAAAASAAASAES<br>GQQGPGGYGPGQQGPGGYGPGQQGPGGYGPGQQGPSGPGSA<br>AAAAAAASGPGQQGPGGYGPGQQGPGGYGPGQQGPSGPGSA<br>AAAAAAASGPGQQGPGGYGPGQQGPGGYGPGQQGLSGPGSA<br>AAAAAAGPGQQGPGGYGPGQQGPSGPGSAAAAAAAAAAGPG	

			GYGPGQQGPGGYGPGQQGPSGAGSAAAAAAAGPGQQGLGG<br>YGPGQQGPGGYGPGQQGPGGYGPGSASAAAAAAGPGQQGPG<br>GYGPGQQGPSGPGSASAAAAAAAAAGPGGYGPGQQGPGGYAP<br>GQQGPSGPGSASAAAAAAAAGPGGYGPGQQGPGGYAPGQQ<br>GPSGPGSAAAAAAAAAGPGGYGPAQQGPSGPGIAASAASAGP<br>GGYGPAQQGPAGYGPGSAVAASAGAGSAGYGPGSQASAAAS<br>RLASPDSGARVASAVSNLVSSGPTSSAALSSVISNAVSQIGASNP<br>GLSGCDVLIQALLEIVSACVTILSSSSIGQVNYGAASQFAQVVGQ<br>SVLSAF	
FLYS,<br>$FLYS_3$<br>$FLYS_4$	*Trichonephila clavipes*	*E. coli*	Module repeated up to 4 times<br>GPGGPGGYGPGGSGPGGYGPGGSGPGGYGPGGSGPGGYGPGG<br>SGPSGPGSAAAAAAAAGPGGYGPGGSGPGGYGPGGSGPGGYG<br>PGGSGPGGYGPGGSGPGGYGPGGSGPSGPGSAAAAAAAAGPG<br>GYGPGGSGPGGYGPGGSGPGGYGPGGSGPSGPGSAAAAAAAA<br>GPGGYGPGGSGPGGYGPGGSGPGGYGPGGSGPGGYGPGGSGP<br>GGYGPGGSGPGGYGPGGSGPSGPGSAAAAAAAAGPGGYGPG<br>GSGPGGYGPGGSGPGGYGPGGSGPGGYGPGGSGPGGYGPGGS<br>GPGGYGPGGSGPGGYGPGGSGPGGYGPGGSGPSGPGSAAAAA<br>AAA	[44,47]
1-mer until 192-mer	*Trichonephila clavipes*	*E. coli*	1 mer module repeated up to 96mer<br>SGRGGLGGQGAGAAAAAGGAGQGGYGGLGSQGT<br>+ cell adhesion peptides (e.g. RGD)<br>+ antimicrobial peptides<br>+ biomineralization peptides<br>+ cancer treating peptides	[49-72]

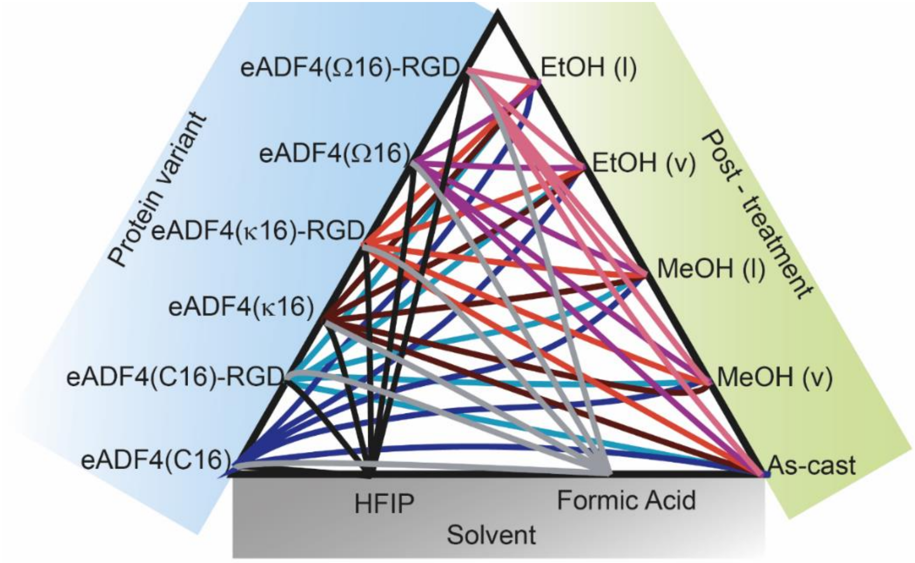

Figure S1. Scheme of the sample matrix of experimental settings used to analyze how the spider silk protein sequence, the casting solvent and the post-treatment influence cell adhesion. The recombinant ADF4 variants (eADF4(C16), eADF4(κ16), eADF4(Ω16), eADF4(C16)-RGD, eADF4(κ16)-RGD and eADF4(Ω16)-RGD) were either solved in FA or HFIP and cast on ozone-pre-treated PS surfaces. Furthermore, coatings were differently post-treated (MeOH vapor or liquid, EtOH vapor or liquid) and compared to non-post-treated films regarding adhesion of human BJ skin fibroblasts and human MG63 bone fibroblasts. The results thereof are summarized in Figure 6, S2 and S3.

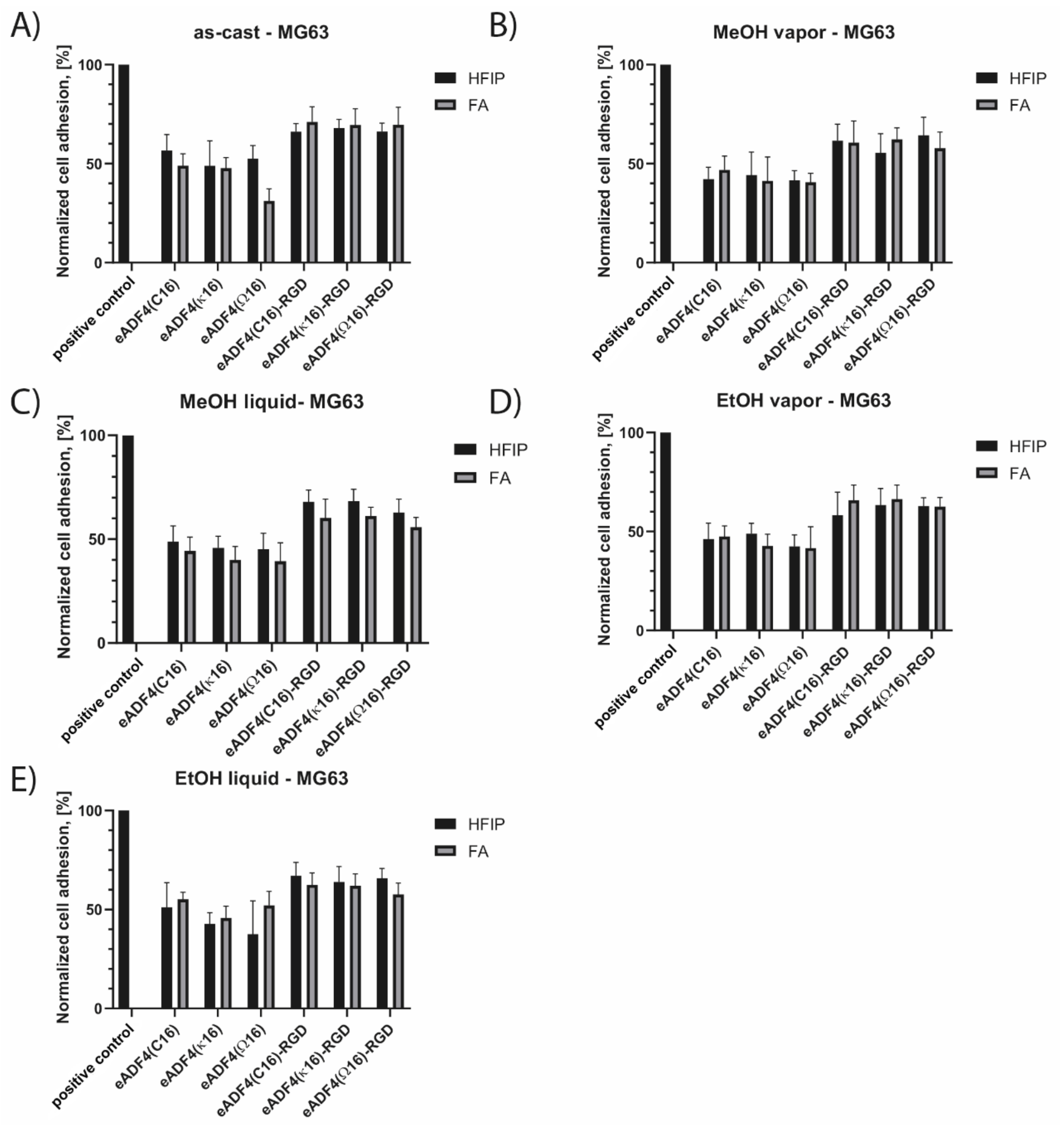

Figure S2. Cell adhesion of MG63 on films made of eADF4 variants (**A**) as cast or post-treated with (**B**) MeOH vapor, (**C**) MeOH liquid, (**D**) EtOH vapor, and (**E**) EtOH liquid.

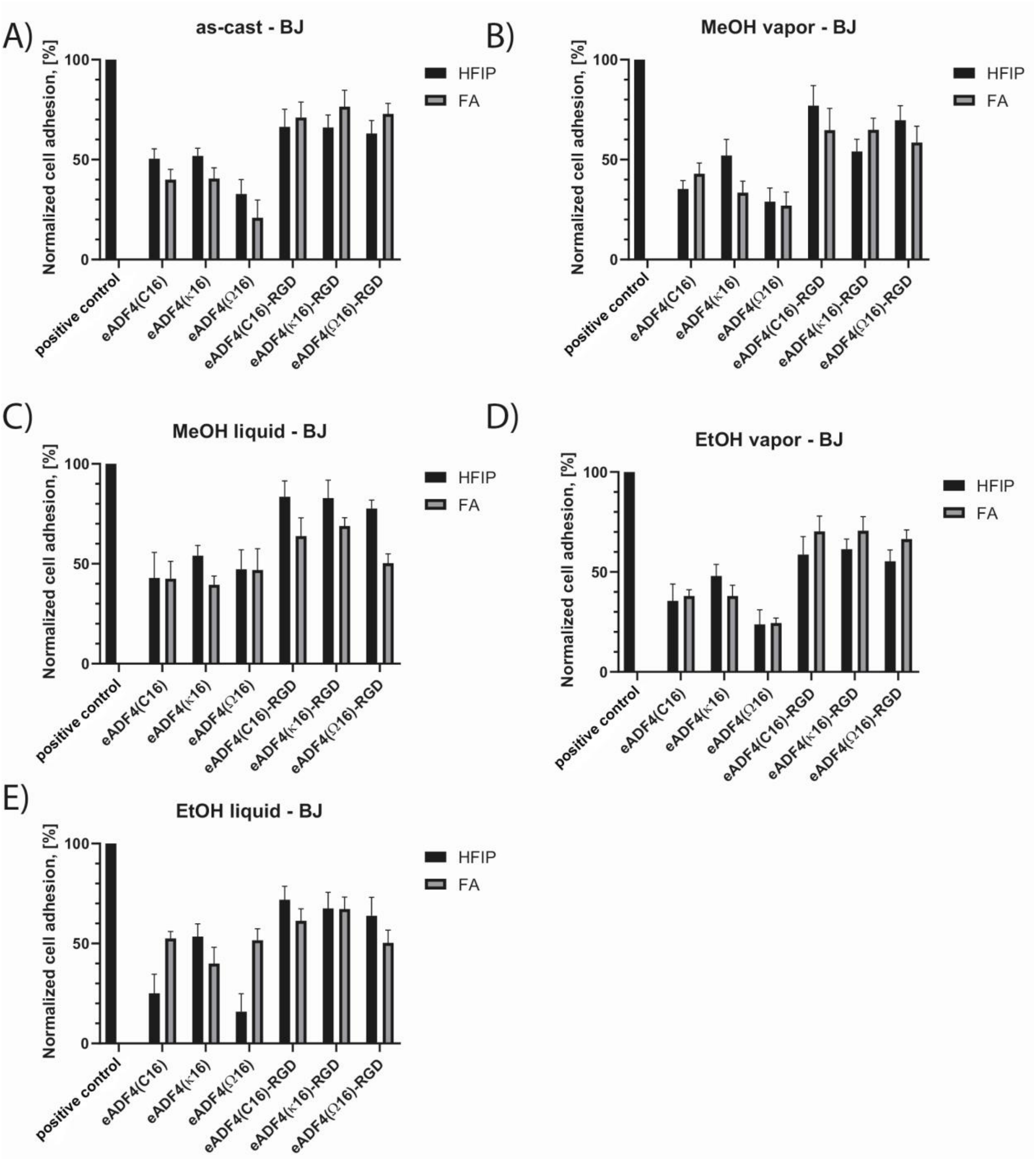

Figure S3. Cell adhesion on films made of BJ of eADF4 variants (**A**) as cast or post-treated with (**B**) MeOH vapor, (**C**) MeOH liquid, (**D**) EtOH vapor, (**E**) EtOH liquid.

References

1. Lentz, S.; Trossmann, V.T.; Borkner, C.B.; Beyersdorfer, V.; Rottmar, M.; Scheibel, T. Structure–Property Relationship Based on the Amino Acid Composition of Recombinant Spider Silk Proteins for Potential Biomedical Applications. *ACS Applied Materials & Interfaces* **2022**, *14*, 31751-31766, doi:10.1021/acsami.2c09590.
2. Kumari, S.; Lang, G.; DeSimone, E.; Spengler, C.; Trossmann, V.T.; Lücker, S.; Hudel, M.; Jacobs, K.; Krämer, N.; Scheibel, T. Engineered spider silk-based 2D and 3D materials prevent microbial infestation. *Materials Today* **2020**, *41*, 21-33, doi:https://doi.org/10.1016/j.mattod.2020.06.009.
3. Esser, T.U.; Trossmann, V.T.; Lentz, S.; Engel, F.B.; Scheibel, T. Designing of spider silk proteins for human induced pluripotent stem cell-based cardiac tissue engineering. *Materials Today Bio* **2021**, *11*, 100114, doi:https://doi.org/10.1016/j.mtbio.2021.100114.
4. Trossmann, V.T.; Scheibel, T. Design of Recombinant Spider Silk Proteins for Cell Type Specific Binding. *Advanced Healthcare Materials* **2023**, *12*, 2202660, doi:https://doi.org/10.1002/adhm.202202660.

5. Borkner, C.B.; Wohlrab, S.; Möller, E.; Lang, G.; Scheibel, T. Surface Modification of Polymeric Biomaterials Using Recombinant Spider Silk Proteins. *ACS Biomaterials Science & Engineering* **2017**, *3*, 767-775, doi:10.1021/acsbiomaterials.6b00306.
6. Herold, H.M.; Döbl, A.; Wohlrab, S.; Humenik, M.; Scheibel, T. Designed Spider Silk-Based Drug Carrier for Redox- or pH-Triggered Drug Release. *Biomacromolecules* **2020**, *21*, 4904-4912, doi:10.1021/acs.biomac.0c01138.
7. Neubauer, V.J.; Scheibel, T. Spider Silk Fusion Proteins for Controlled Collagen Binding and Biomineralization. *ACS Biomater Sci Eng* **2020**, *6*, 5599-5608, doi:10.1021/acsbiomaterials.0c00818.
8. Mohammadi, P.; Aranko, A.S.; Lemetti, L.; Cenev, Z.; Zhou, Q.; Virtanen, S.; Landowski, C.P.; Penttilä, M.; Fischer, W.J.; Wagermaier, W.; et al. Phase transitions as intermediate steps in the formation of molecularly engineered protein fibers. *Communications Biology* **2018**, *1*, 86, doi:10.1038/s42003-018-0090-y.
9. Välisalmi, T.; Roas-Escalona, N.; Meinander, K.; Mohammadi, P.; Linder, M.B. Highly Hydrophobic Films of Engineered Silk Proteins by a Simple Deposition Method. *Langmuir* **2023**, *39*, 4370-4381, doi:10.1021/acs.langmuir.2c03442.
10. Mohammadi, P.; Aranko, A.S.; Landowski, C.P.; Ikkala, O.; Jaudzems, K.; Wagermaier, W.; Linder, M.B. Biomimetic composites with enhanced toughening using silk-inspired triblock proteins and aligned nanocellulose reinforcements. *Science advances* **2019**, *5*, eaaw2541.
11. Hedhammar, M.; Rising, A.; Grip, S.; Martinez, A.S.; Nordling, K.; Casals, C.; Stark, M.; Johansson, J. Structural Properties of Recombinant Nonrepetitive and Repetitive Parts of Major Ampullate Spidroin 1 from Euprosthenops australis: Implications for Fiber Formation. *Biochemistry* **2008**, *47*, 3407-3417, doi:10.1021/bi702432y.
12. Stark, M.; Grip, S.; Rising, A.; Hedhammar, M.; Engström, W.; Hjälm, G.; Johansson, J. Macroscopic Fibers Self-Assembled from Recombinant Miniature Spider Silk Proteins. *Biomacromolecules* **2007**, *8*, 1695-1701, doi:10.1021/bm070049y.
13. Widhe, M.; Shalaly, N.D.; Hedhammar, M. A fibronectin mimetic motif improves integrin mediated cell biding to recombinant spider silk matrices. *Biomaterials* **2016**, *74*, 256-266, doi:https://doi.org/10.1016/j.biomaterials.2015.10.013.
14. Widhe, M.; Johansson, U.; Hillerdahl, C.-O.; Hedhammar, M. Recombinant spider silk with cell binding motifs for specific adherence of cells. *Biomaterials* **2013**, *34*, 8223-8234, doi:https://doi.org/10.1016/j.biomaterials.2013.07.058.
15. Chouhan, D.; Thatikonda, N.; Nilebäck, L.; Widhe, M.; Hedhammar, M.; Mandal, B.B. Recombinant Spider Silk Functionalized Silkworm Silk Matrices as Potential Bioactive Wound Dressings and Skin Grafts. *ACS Applied Materials & Interfaces* **2018**, *10*, 23560-23572, doi:10.1021/acsami.8b05853.
16. Nilebäck, L.; Hedin, J.; Widhe, M.; Floderus, L.S.; Krona, A.; Bysell, H.; Hedhammar, M. Self-Assembly of Recombinant Silk as a Strategy for Chemical-Free Formation of Bioactive Coatings: A Real-Time Study. *Biomacromolecules* **2017**, *18*, 846-854, doi:10.1021/acs.biomac.6b01721.
17. Jansson, R.; Thatikonda, N.; Lindberg, D.; Rising, A.; Johansson, J.; Nygren, P.-Å.; Hedhammar, M. Recombinant Spider Silk Genetically Functionalized with Affinity Domains. *Biomacromolecules* **2014**, *15*, 1696-1706, doi:10.1021/bm500114e.
18. Thatikonda, N.; Nilebäck, L.; Kempe, A.; Widhe, M.; Hedhammar, M. Bioactivation of Spider Silk with Basic Fibroblast Growth Factor for in Vitro Cell Culture: A Step toward Creation of Artificial ECM. *ACS Biomaterials Science & Engineering* **2018**, *4*, 3384-3396, doi:10.1021/acsbiomaterials.8b00844.
19. Jansson, R.; Courtin, C.M.; Sandgren, M.; Hedhammar, M. Rational Design of Spider Silk Materials Genetically Fused with an Enzyme. *Advanced Functional Materials* **2015**, *25*, 5343-5352, doi:https://doi.org/10.1002/adfm.201501833.
20. Nilebäck, L.; Chouhan, D.; Jansson, R.; Widhe, M.; Mandal, B.B.; Hedhammar, M. Silk–Silk Interactions between Silkworm Fibroin and Recombinant Spider Silk Fusion Proteins Enable the Construction of Bioactive Materials. *ACS Applied Materials & Interfaces* **2017**, *9*, 31634-31644, doi:10.1021/acsami.7b10874.
21. Nilebäck, L.; Widhe, M.; Seijsing, J.; Bysell, H.; Sharma, P.K.; Hedhammar, M. Bioactive Silk Coatings Reduce the Adhesion of Staphylococcus aureus while Supporting Growth of Osteoblast-like Cells. *ACS Applied Materials & Interfaces* **2019**, *11*, 24999-25007, doi:10.1021/acsami.9b05531.
22. Tasiopoulos, C.P.; Petronis, S.; Sahlin, H.; Hedhammar, M. Surface Functionalization of PTFE Membranes Intended for Guided Bone Regeneration Using Recombinant Spider Silk. *ACS Appl Bio Mater* **2020**, *3*, 577-583, doi:10.1021/acsabm.9b00972.
23. Jansson, R.; Lau, C.H.; Ishida, T.; Ramström, M.; Sandgren, M.; Hedhammar, M. Functionalized silk assembled from a recombinant spider silk fusion protein (Z-4RepCT) produced in the methylotrophic yeast Pichia pastoris. *Biotechnol J* **2016**, *11*, 687-699, doi:10.1002/biot.201500412.

24. Hedhammar, M.; Bramfeldt, H.; Baris, T.; Widhe, M.; Askarieh, G.; Nordling, K.; von Aulock, S.; Johansson, J. Sterilized Recombinant Spider Silk Fibers of Low Pyrogenicity. *Biomacromolecules* **2010**, *11*, 953-959, doi:10.1021/bm9014039.
25. Gustafsson, L.; Jansson, R.; Hedhammar, M.; van der Wijngaart, W. Structuring of Functional Spider Silk Wires, Coatings, and Sheets by Self-Assembly on Superhydrophobic Pillar Surfaces. *Adv Mater* **2018**, *30*, doi:10.1002/adma.201704325.
26. Nilebäck, L.; Arola, S.; Kvick, M.; Paananen, A.; Linder, M.B.; Hedhammar, M. Interfacial Behavior of Recombinant Spider Silk Protein Parts Reveals Cues on the Silk Assembly Mechanism. *Langmuir* **2018**, *34*, 11795-11805, doi:10.1021/acs.langmuir.8b02381.
27. Chouhan, D.; Lohe, T.-u.; Thatikonda, N.; Naidu, V.G.M.; Hedhammar, M.; Mandal, B.B. Silkworm Silk Scaffolds Functionalized with Recombinant Spider Silk Containing a Fibronectin Motif Promotes Healing of Full-Thickness Burn Wounds. *ACS Biomaterials Science & Engineering* **2019**, *5*, 4634-4645, doi:10.1021/acsbiomaterials.9b00887.
28. Widhe, M.; Bysell, H.; Nystedt, S.; Schenning, I.; Malmsten, M.; Johansson, J.; Rising, A.; Hedhammar, M. Recombinant spider silk as matrices for cell culture. *Biomaterials* **2010**, *31*, 9575-9585, doi:https://doi.org/10.1016/j.biomaterials.2010.08.061.
29. Gustafsson, L.; Tasiopoulos, C.P.; Jansson, R.; Kvick, M.; Duursma, T.; Gasser, T.C.; van der Wijngaart, W.; Hedhammar, M. Recombinant Spider Silk Forms Tough and Elastic Nanomembranes that are Protein-Permeable and Support Cell Attachment and Growth. *Advanced Functional Materials* **2020**, *30*, 2002982, doi:https://doi.org/10.1002/adfm.202002982.
30. Lewicka, M.; Hermanson, O.; Rising, A.U. Recombinant spider silk matrices for neural stem cell cultures. *Biomaterials* **2012**, *33*, 7712-7717, doi:https://doi.org/10.1016/j.biomaterials.2012.07.021.
31. Fredriksson, C.; Hedhammar, M.; Feinstein, R.; Nordling, K.; Kratz, G.; Johansson, J.; Huss, F.; Rising, A. Tissue response to subcutaneously implanted recombinant spider silk: an in vivo study. *Materials* **2009**, *2*, 1908-1922.
32. Lewicka, M.; Rebellato, P.; Lewicki, J.; Uhlén, P.; Rising, A.; Hermanson, O. Recombinant Spider Silk Protein Matrices Facilitate Differentiation of Neural Stem Cells Into Mature and Functional Neurons. *Frontiers in Materials* **2021**, *7*, doi:10.3389/fmats.2020.560372.
33. Johansson, U.; Ria, M.; Åvall, K.; Dekki Shalaly, N.; Zaitsev, S.V.; Berggren, P.-O.; Hedhammar, M. Pancreatic Islet Survival and Engraftment Is Promoted by Culture on Functionalized Spider Silk Matrices. *PLOS ONE* **2015**, *10*, e0130169, doi:10.1371/journal.pone.0130169.
34. Shalaly, N.D.; Ria, M.; Johansson, U.; Åvall, K.; Berggren, P.-O.; Hedhammar, M. Silk matrices promote formation of insulin-secreting islet-like clusters. *Biomaterials* **2016**, *90*, 50-61, doi:https://doi.org/10.1016/j.biomaterials.2016.03.006.
35. Thatikonda, N.; Delfani, P.; Jansson, R.; Petersson, L.; Lindberg, D.; Wingren, C.; Hedhammar, M. Genetic fusion of single-chain variable fragments to partial spider silk improves target detection in micro- and nanoarrays. *Biotechnology Journal* **2016**, *11*, 437-448, doi:https://doi.org/10.1002/biot.201500297.
36. Grip, S.; Johansson, J.; Hedhammar, M. Engineered disulfides improve mechanical properties of recombinant spider silk. *Protein Sci* **2009**, *18*, 1012-1022, doi:10.1002/pro.111.
37. Leppert, A.; Chen, G.; Lama, D.; Sahin, C.; Railaite, V.; Shilkova, O.; Arndt, T.; Marklund, E.G.; Lane, D.P.; Rising, A.; et al. Liquid–Liquid Phase Separation Primes Spider Silk Proteins for Fiber Formation via a Conditional Sticker Domain. *Nano Letters* **2023**, *23*, 5836-5841, doi:10.1021/acs.nanolett.3c00773.
38. Andersson, M.; Jia, Q.; Abella, A.; Lee, X.-Y.; Landreh, M.; Purhonen, P.; Hebert, H.; Tenje, M.; Robinson, C.V.; Meng, Q.; et al. Biomimetic spinning of artificial spider silk from a chimeric minispidroin. *Nature Chemical Biology* **2017**, *13*, 262-264, doi:10.1038/nchembio.2269.
39. Arndt, T.; Greco, G.; Schmuck, B.; Bunz, J.; Shilkova, O.; Francis, J.; Pugno, N.M.; Jaudzems, K.; Barth, A.; Johansson, J.; et al. Engineered Spider Silk Proteins for Biomimetic Spinning of Fibers with Toughness Equal to Dragline Silks. *Advanced Functional Materials* **2022**, *32*, 2200986, doi:https://doi.org/10.1002/adfm.202200986.
40. Schmuck, B.; Greco, G.; Bäcklund, F.G.; Pugno, N.M.; Johansson, J.; Rising, A. Impact of physio-chemical spinning conditions on the mechanical properties of biomimetic spider silk fibers. *Communications Materials* **2022**, *3*, 83, doi:10.1038/s43246-022-00307-6.
41. Liu, F.Y.C. Silk-based antimicrobial peptide mixed with recombinant spidroin creates functionalized spider silk. *bioRxiv* **2021**, 2021.2003.2026.437269, doi:10.1101/2021.03.26.437269.
42. Jones, J.A.; Harris, T.I.; Bell, B.E.; Oliveira, P.F. Material Formation of Recombinant Spider Silks through Aqueous Solvation using Heat and Pressure. *J Vis Exp* **2019**, doi:10.3791/59318.
43. Jones, J.A.; Harris, T.I.; Tucker, C.L.; Berg, K.R.; Christy, S.Y.; Day, B.A.; Gaztambide, D.A.; Needham, N.J.C.; Ruben, A.L.; Oliveira, P.F.; et al. More Than Just Fibers: An Aqueous Method for the Production of Innovative

Recombinant Spider Silk Protein Materials. *Biomacromolecules* **2015**, *16*, 1418-1425, doi:https://doi.org/10.1021/acs.biomac.5b00226.

44. Harris, T.I.; Gaztambide, D.A.; Day, B.A.; Brock, C.L.; Ruben, A.L.; Jones, J.A.; Lewis, R.V. Sticky Situation: An Investigation of Robust Aqueous-Based Recombinant Spider Silk Protein Coatings and Adhesives. *Biomacromolecules* **2016**, *17*, 3761-3772, doi:10.1021/acs.biomac.6b01267.
45. Tucker, C.L.; Jones, J.A.; Bringhurst, H.N.; Copeland, C.G.; Addison, J.B.; Weber, W.S.; Mou, Q.; Yarger, J.L.; Lewis, R.V. Mechanical and Physical Properties of Recombinant Spider Silk Films Using Organic and Aqueous Solvents. *Biomacromolecules* **2014**, *15*, 3158-3170, doi:10.1021/bm5007823.
46. An, B.; Tang-Schomer, M.; Huang, W.; He, J.; Jones, J.; Lewis, R.V.; Kaplan, D.L. Physical and biological regulation of neuron regenerative growth and network formation on recombinant dragline silks. *Biomaterials* **2015**, *48*, 137-146, doi:10.1016/j.biomaterials.2015.01.044.
47. Bhattacharyya, G.; Oliveira, P.; Krishnaji, S.T.; Chen, D.; Hinman, M.; Bell, B.; Harris, T.I.; Ghazitabatabaei, A.; Lewis, R.V.; Jones, J.A. Large scale production of synthetic spider silk proteins in Escherichia coli. *Protein Expression and Purification* **2021**, *183*, 105839, doi:https://doi.org/10.1016/j.pep.2021.105839.
48. Decker, R.E.; Harris, T.I.; Memmott, D.R.; Peterson, C.J.; Lewis, R.V.; Jones, J.A. Method for the Destruction of Endotoxin in Synthetic Spider Silk Proteins. *Sci Rep* **2018**, *8*, 12166, doi:10.1038/s41598-018-29719-6.
49. Franco, A.R.; Fernandes, E.M.; Rodrigues, M.T.; Rodrigues, F.J.; Gomes, M.E.; Leonor, I.B.; Kaplan, D.L.; Reis, R.L. Antimicrobial coating of spider silk to prevent bacterial attachment on silk surgical sutures. *Acta Biomaterialia* **2019**, *99*, 236-246, doi:https://doi.org/10.1016/j.actbio.2019.09.004.
50. Franco, A.R.; Palma Kimmerling, E.; Silva, C.; Rodrigues, F.J.; Leonor, I.B.; Reis, R.L.; Kaplan, D.L. Silk-Based Antimicrobial Polymers as a New Platform to Design Drug-Free Materials to Impede Microbial Infections. *Macromolecular Bioscience* **2018**, *18*, 1800262, doi:https://doi.org/10.1002/mabi.201800262.
51. Gomes, S.; Gallego - Llamas, J.; Leonor, I.B.; Mano, J.F.; Reis, R.L.; Kaplan, D.L. Biological responses to spider silk - antibiotic fusion protein. *Journal of tissue engineering and regenerative medicine* **2012**, *6*, 356-368.
52. Bini, E.; Foo, C.W.P.; Huang, J.; Karageorgiou, V.; Kitchel, B.; Kaplan, D.L. RGD-Functionalized Bioengineered Spider Dragline Silk Biomaterial. *Biomacromolecules* **2006**, *7*, 3139-3145, doi:10.1021/bm0607877.
53. Xia, X.X.; Qian, Z.G.; Ki, C.S.; Park, Y.H.; Kaplan, D.L.; Lee, S.Y. Native-sized recombinant spider silk protein produced in metabolically engineered Escherichia coli results in a strong fiber. *Proc Natl Acad Sci U S A* **2010**, *107*, 14059-14063, doi:10.1073/pnas.1003366107.
54. Peng, X.; Cui, Y.; Chen, J.; Gao, C.; Yang, Y.; Yu, W.; Rai, K.; Zhang, M.; Nian, R.; Bao, Z.; et al. High-Strength Collagen-Based Composite Films Regulated by Water-Soluble Recombinant Spider Silk Proteins and Water Annealing. *ACS Biomaterials Science & Engineering* **2022**, *8*, 3341-3353, doi:10.1021/acsbiomaterials.2c00416.
55. Peng, Q.; Zhang, Y.; Lu, L.; Shao, H.; Qin, K.; Hu, X.; Xia, X. Recombinant spider silk from aqueous solutions via a bio-inspired microfluidic chip. *Sci Rep* **2016**, *6*, 36473, doi:10.1038/srep36473.
56. Rabotyagova, O.S.; Cebe, P.; Kaplan, D.L. Self-Assembly of Genetically Engineered Spider Silk Block Copolymers. *Biomacromolecules* **2009**, *10*, 229-236, doi:10.1021/bm800930x.
57. Numata, K.; Mieszawska-Czajkowska, A.J.; Kvenvold, L.A.; Kaplan, D.L. Silk-Based Nanocomplexes with Tumor-Homing Peptides for Tumor-Specific Gene Delivery. *Macromolecular Bioscience* **2012**, *12*, 75-82, doi:https://doi.org/10.1002/mabi.201100274.
58. Numata, K.; Reagan, M.R.; Goldstein, R.H.; Rosenblatt, M.; Kaplan, D.L. Spider Silk-Based Gene Carriers for Tumor Cell-Specific Delivery. *Bioconjugate Chemistry* **2011**, *22*, 1605-1610, doi:10.1021/bc200170u.
59. Bowen, C.H.; Dai, B.; Sargent, C.J.; Bai, W.; Ladiwala, P.; Feng, H.; Huang, W.; Kaplan, D.L.; Galazka, J.M.; Zhang, F. Recombinant Spidroins Fully Replicate Primary Mechanical Properties of Natural Spider Silk. *Biomacromolecules* **2018**, *19*, 3853-3860, doi:https://doi.org/10.1021/acs.biomac.8b00980.
60. Dinjaski, N.; Plowright, R.; Zhou, S.; Belton, D.J.; Perry, C.C.; Kaplan, D.L. Osteoinductive recombinant silk fusion proteins for bone regeneration. *Acta Biomater* **2017**, *49*, 127-139, doi:10.1016/j.actbio.2016.12.002.
61. Wong Po Foo, C.; Patwardhan, S.V.; Belton, D.J.; Kitchel, B.; Anastasiades, D.; Huang, J.; Naik, R.R.; Perry, C.C.; Kaplan, D.L. Novel nanocomposites from spider silk–silica fusion (chimeric) proteins. *Proceedings of the National Academy of Sciences* **2006**, *103*, 9428-9433, doi:doi:10.1073/pnas.0601096103.
62. Huang, J.; Wong, C.; George, A.; Kaplan, D.L. The effect of genetically engineered spider silk-dentin matrix protein 1 chimeric protein on hydroxyapatite nucleation. *Biomaterials* **2007**, *28*, 2358-2367, doi:https://doi.org/10.1016/j.biomaterials.2006.11.021.
63. Meirovitch, S.; Shtein, Z.; Ben-Shalom, T.; Lapidot, S.; Tamburu, C.; Hu, X.; Kluge, J.; Raviv, U.; Kaplan, D.; Shoseyov, O. Spider Silk-CBD-Cellulose Nanocrystal Composites: Mechanism of Assembly. *International journal of molecular sciences* **2016**, *17*, doi:10.3390/ijms17091573.
64. Gomes, S.C.; Leonor, I.B.; Mano, J.F.; Reis, R.L.; Kaplan, D.L. Antimicrobial functionalized genetically engineered spider silk. *Biomaterials* **2011**, *32*, 4255-4266, doi:https://doi.org/10.1016/j.biomaterials.2011.02.040.

65. Gomes, S.; Gallego-Llamas, J.; Leonor, I.B.; Mano, J.F.; Reis, R.L.; Kaplan, D.L. In Vivo Biological Responses to Silk Proteins Functionalized with Bone Sialoprotein. *Macromolecular Bioscience* **2013**, *13*, 444-454, doi:https://doi.org/10.1002/mabi.201200372.
66. Gomes, S.; Leonor, I.B.; Mano, J.F.; Reis, R.L.; Kaplan, D.L. Spider silk-bone sialoprotein fusion proteins for bone tissue engineering. *Soft Matter* **2011**, *7*, 4964-4973, doi:10.1039/C1SM05024A.
67. Numata, K.; Kaplan, D.L. Silk-Based Gene Carriers with Cell Membrane Destabilizing Peptides. *Biomacromolecules* **2010**, *11*, 3189-3195, doi:10.1021/bm101055m.
68. Plowright, R.; Dinjaski, N.; Zhou, S.; Belton, D.J.; Kaplan, D.L.; Perry, C.C. Influence of silk–silica fusion protein design on silica condensation in vitro and cellular calcification. *RSC Advances* **2016**, *6*, 21776-21788, doi:10.1039/C6RA03706B.
69. Belton, D.J.; Mieszawska, A.J.; Currie, H.A.; Kaplan, D.L.; Perry, C.C. Silk-silica composites from genetically engineered chimeric proteins: materials properties correlate with silica condensation rate and colloidal stability of the proteins in aqueous solution. *Langmuir* **2012**, *28*, 4373-4381, doi:10.1021/la205084z.
70. Zhou, S.; Huang, W.; Belton, D.J.; Simmons, L.O.; Perry, C.C.; Wang, X.; Kaplan, D.L. Control of silicification by genetically engineered fusion proteins: silk-silica binding peptides. *Acta Biomater* **2015**, *15*, 173-180, doi:10.1016/j.actbio.2014.10.040.
71. Numata, K.; Subramanian, B.; Currie, H.A.; Kaplan, D.L. Bioengineered silk protein-based gene delivery systems. *Biomaterials* **2009**, *30*, 5775-5784, doi:10.1016/j.biomaterials.2009.06.028.
72. Mieszawska, A.J.; Nadkarni, L.D.; Perry, C.C.; Kaplan, D.L. Nanoscale control of silica particle formation via silk-silica fusion proteins for bone regeneration. *Chem Mater* **2010**, *22*, 5780-5785, doi:10.1021/cm101940u.
